# Integrated Pharmacogenetics Analysis of the Three Fangjis Decoctions for Treating Arrhythmias Based on Molecular Network Patterns

**DOI:** 10.3389/fcvm.2021.726694

**Published:** 2021-12-24

**Authors:** Penglu Wei, Dehuai Long, Yupei Tan, Wenlong Xing, Xiang Li, Kuo Yang, Hongxu Liu

**Affiliations:** ^1^Beijing Hospital of Traditional Chinese Medicine, Capital Medical University, Beijing, China; ^2^Beijing University of Chinese Medicine, Beijing, China; ^3^School of Computer and Information Technology, Institute of Medical Intelligence, Beijing Jiaotong University, Beijing, China

**Keywords:** fangjis, arrhythmias, target distribution, network pharmacology, precision medicine

## Abstract

**Aim:** To explore the diverse target distribution and variable mechanisms of different fangjis prescriptions when treating arrhythmias based on the systems pharmacology.

**Methods:** The active ingredients and their corresponding targets were acquired from the three fangjis [Zhigancao Tang (ZT), Guizhigancao Longgumuli Tang (GLT), and Huanglian E'jiao Tang (HET)] and the arrhythmia-related genes were identified based on comprehensive database screening. Networks were constructed between the fangjis and arrhythmia and used to define arrhythmia modules. Common and differential gene targets were identified within the arrhythmia network modules and the cover rate (CR) matrix was applied to compare the contributions of the fangjis to the network and modules. Comparative pharmacogenetics analyses were then conducted to define the arrhythmia-related signaling pathways regulated by the fangjis prescriptions. Finally, the divergence and convergence points of the arrhythmia pathways were deciphered based on databases and the published literature.

**Results:** A total of 187, 105, and 68 active ingredients and 1,139, 1,195, and 811 corresponding gene targets of the three fangjis were obtained and 102 arrhythmia-related genes were acquired. An arrhythmia network was constructed and subdivided into 4 modules. For the target distribution analysis, 65.4% of genes were regulated by the three fangjis within the arrhythmia network. ZT and GLT were more similar to each other, mainly regulated by module two, whereas HET was divided among all the modules. From the perspective of signal transduction, calcium-related pathways [calcium, cyclic guanosine 3′,5′-monophosphate (cGMP)-PKG, and cyclic adenosine 3′,5′-monophosphate (cAMP)] and endocrine system-related pathways (oxytocin signaling pathway and renin secretion pathways) were associated with all the three fangjis prescriptions. Nevertheless, heterogeneity existed between the biological processes and pathway distribution among the three prescriptions. GLT and HET were particularly inclined toward the conditions involving abnormal hormone secretion, whereas ZT tended toward renin-angiotensin-aldosterone system (RAAS) disorders. However, calcium signaling-related pathways prominently feature in the pharmacological activities of the decoctions. Experimental validation indicated that ZT, GLT, and HET significantly shortened the duration of ventricular arrhythmia (VA) and downregulated the expression of CALM2 and interleukin-6 (IL-6) messenger RNAs (mRNAs); GLT and HET downregulated the expression of CALM1 and NOS3 mRNAs; HET downregulated the expression of CRP mRNA.

**Conclusion:** Comparing the various distributions of the three fangjis, pathways provide evidence with respect to precise applications toward individualized arrhythmia treatments.

## Introduction

Cardiovascular disease (CVD), which includes arrhythmia, has long been recognized as the major cause of global death and morbidity. Epidemiological studies have shown that atrial fibrillation (AF) affects 1–2% of the population and may reach unprecedented levels as populations around the world age (1, 2). Arrhythmia is a complex disease, resulting from the interactions between multiple genetic and environmental factors (3). Indirect pieces of evidence using molecular phenotyping, cluster analysis, and biomarker study suggest that heterogeneity generally exists within arrhythmias (4, 5). Therefore, different regimens may be necessary to adequately treat diverse phenotypes among arrhythmias. Combination therapy is a promising strategy against changeable and complex disorders. Fangjis consist of well-designed decoctions of different herbs, which aim to optimize the therapeutic effects and/or reduce toxicity (6).

Notably, fangjis prescriptions have a long history of use and their effectiveness has been previously verified in clinical settings (7–9). Fangjis possess “multitarget, multipathway” traits, which are not only beneficial in combatting complex diseases and flexible phenotypes, but also allow personalized treatment plans to be designed in accordance with genetic characteristics of an individual patient. Of these, Zhigancao Tang (ZT) (10, 11), Guizhigancao Longgumuli Tang (GLT) (12), and Huanglian E'jiao Tang (HET) (13) are classical formulae commonly applied against arrhythmias and can be directed toward different clinical phenotypes (syndromes).

The crucial question is how to accurately identify the target distribution for each of the fangjis preparations and to match these precisely to the corresponding treatment population (14).

Systems pharmacology builds a paradigm for drug–target interactions, which enables a better understanding of the mechanism of action of drugs at the molecular level (15, 16). Furthermore, advances in sequencing techniques, combinatorial chemistry, genome-wide association studies (GWASs), and other large-scale omics (genomics, transcriptomics, metabolomics, and proteomics) methodologies at different biological levels (molecular, cellular, tissue, and organism), multilayer computational networks, and subnetworks (17, 18) can be integrated to help decrypt complex diseases (19, 20). Network-based precision medicine has made substantial progress, especially in areas such as cancer research (21). Previous studies deciphered the different mechanisms of drug combinations (different components of Qingkailing) based on omics data through the analysis of cerebral ischemia pathways profile (22, 23), but phenotype-dependent association analysis is presently insufficient. In this study, we selected the three fangjis (ZT, GLT, and HET) specific to different syndromes and ascertained the target distribution and functional enrichment based on pharmacogenetics data and molecular network modeling. Together this, it provides evidence-based support for precision application of the fangjis.

## Materials and Methods

### Data Source of the Fangjis and Disease

The constituent herbs of these three classical fangjis including ZT, GLT, and HET are given in Table 1. The ingredients and corresponding targets were selected using the Traditional Chinese Medicine Systems Pharmacology Database and Analysis Platform (TCMSP) (http://lsp.nwu.edu.cn/tcmsp.php) and the TCM mesh database (http://mesh.tcm.microbioinformatics.org/). Arrhythmia-related genes were acquired from the National Center for Biotechnology Information (NCBI) (https://www.ncbi.nlm.nih.gov/) database, using the search terms “arrhythmia” with “*Homo sapiens*” set as the background.

**Table 1:** The constituent herbs of the three fangjis.

**Fangjis**	**Traditional efficacy**	**References**	**Herbs**	**English name**	**Latin name**	**Properties**	**Merdians**	**Sources**	**Species**	**Part used**	**Dosage/g**
Zhigancao Tang (ZT)	nourishing yin and activating yang	(10, 11)	Gancao	Liquorice	Glycyrrhizae Radix et Rhizoma	Mild, Sweet	Lung, Spleen, Stomach, Heart	Glycyrrhiza uralensis Fisc.; Glycyrrhiza inflata Bat; Glycyrrhiza glabra L.	Leguminosae	Root and rhizome	2–10g
			Shengjiang	Ginger	Zingiberis Rhizoma Recens	Warm, Pungent	Lung, Spleen, Stomach	Zingiber officinale Rosc.	Zingiberaceae	Fresh rhizome	3–10g
			Renshen	Ginseng	Ginseng Radix et Rhizoma	Minor Warm, Sweet, Slightly Bitter	Lung, Spleen, Heart	Panaxginseng C. A. Mey.	Araliaceae	Root	3–9g
			Shengdihuang	Rehmannia glutinosa	Rehmanniae Radix	Cold, Sweet, Bitter	Liver, Heart, Kidney	Rehmannia glutinosa Libosch.	Scrophulariaceae	Tuberoid	12–30g
			Guizhi	Cassia Twig	Cinnamomi Ramulus	Warm, Sweet, Pungent	Lung, Bladder, Heart	Cinnamomum cassia Presl	Lauraceae	Twig	3–10g
			Ejiao	donkey-hide gelatin	Asini Corii Colla	Mild, Sweet	Lung, Liver, Kidney	Equus asinus L.	Equidae	Skin	3–9g
			Maidong	Ophiopogon japonicus	Ophiopogonis Radix	Minor cold, Sweet	Lung, Stomach, Heart	Ophiopogon japonicus (L.f)Ker-Gawl.	liliaceae	Tuberoid	6–12g
			Huomaren	Fructus Cannabis	Cannabis Fructus	Mild, Sweet	Spleen, Large Intestine, Stomach	Cannabis sativa L.	Moraceae	Ripe seed	10–15g
			Jiu	Liqueur	Vinum	Warm, Sweet, Pungent, Bitter	Lung, Stomach, Liver, Heart	Rice, Wheat, Millet, Sorghum and other koji	Medicinal drinks	Jiu	50–100g
			Dazao	Jujube	Jujubae Fructus	Warm, Sweet	Spleen, Stomach	Ziziphus jujuba Mill.	Rhamnaceae	Ripe fruit	6–15g
Guizhigancao Longgumuli Tang (GLT)	warming yang	(12)	Guizi	Cassia Twig	Cinnamomi Ramulus	Warm, Sweet, Pungent	Lung, Bladder, Heart	Cinnamomum cassia Presl	Lauraceae	Twig	3–10g
			Gancao	Liquorice	Glycyrrhizae Radix et Rhizoma	Mild, Sweet	Lung, Spleen, Stomach, Heart	Glycyrrhiza uralensis Fisch; Glycyrrhiza inflate Bat; Glycyrrhiza glabra L.	Leguminosae	Root and rhizome	2–10g
			Longgu	Keel	Os Draconis	Mild, Sweet, Punkery	Large Intestine, Liver, Heart, Kidney	Elephants, Rhinoceros, Horses.	Mammalia	Fossilized bones	10–15g
			Muli	Oyster	Ostreae Concha	Minor cold, Salty, Punkery	Liver, Kidney	Ostrea gigas Thunberg, Ostrea talienwhanensis Crosse, Ostrea rivularis Gould	Ostreidae	Conch	9–30g
Huangliang E'jiao Tang (HET)	nourishing yin	(13)	Huangqin	Scutellaria baicalensis	Scutellariae Radix	Cold, Bitter	Lung, Large Intestine, Stomach, Small Intestine, Gallbladder	Scutellaria baicalensis Georgi	Lamiaceae	Root	3–10g
			Huanglian	Coptis chinensis	Coptidis Rhizoma	Cold, Bitter	Large Intestine, Stomach, Small Intestine, Liver, Heart	Coptis chinensis Franch., Coptis deltoidea C.Y. Cheng et Hsiao, Coptis teeta Wall.	Ranunculaceae	Rhizome	2–5g
			Baishao	White Paeony Root	Paeoniae Radix Alba	Minor cold, Sour, Bitter	Spleen, Liver	Paeonia lactiflora Pall.	Ranunculaceae	Root	6–15g
			E'jiao	donkey-hide gelatin	Asini Corii Colla	Mild, Sweet	Lung, Liver, Kidney	Equus asinus L.	Equidae	Skin	3–9g
			Jizihuang	Yolk	Gallus gallus domesticus	Mild, Sweet	Lung, Heart, Kidney	Gallus gallus domesticus Brisson	Phasianidae	Egg yolk	1

### Network Construction and Modular Division

The arrhythmia-related genes were uploaded to the Search Tool for Recurring Instances of Neighboring Genes (STRING) (https://string-db.org/) database using the “*Homo sapiens*” background to establish associations between the genes and then to acquire the arrhythmia-related network. The three decoctions of ZT, GLT, and HET were independently mapped to the arrhythmia-related network; the Molecular Complex Detection (MCODE) method was used to conduct cluster analysis in order to discover structural modules based on topology, and their similarities and differences were characterized both in the disease networks and modules.

### Comparison of the Fangjis for Treating Arrhythmia

To observe the distribution of the three fangjis in the disease interaction network and modules, the coverage rate (CR) was defined as follows:


$$\begin{array}{l} CR_{fm}=\frac{X_{fm}}{Y_{m}} \end{array}$$

F represented any formula and *CR*_*fm*_ was the coverage rate of formula f on module m in the arrhythmia-related network; Y_m_ was the overall genes in module m; X_fm_ was the number of targets covered by formula f in module m. Letting f = {f_1_, f_2_, ……, f_n_} be a given set of formulas and m = {m_1_, m_2_, ……, m_n_} be a given set of modules, Y_f_ × Y_m_ was considered as a binary matrix of module-formula association.

### The Gene Ontology (GO) Enrichment and the Kyoto Encyclopedia of Genes and Genomes (KEGG) Signaling Analysis

The online database such as the Database for Annotation, Visualization, and Integrated Discovery (DAVID) was used to perform the GO biological processes and the KEGG pathway analysis. The GO and the KEGG significance levels were both set at a *p*-value, once corrected with the Bonferroni correction, at <0.05.

### Characteristic Targeting Pathways of the Fangjis

According to the results from the bioinformatics analysis and literature search resulted from PubMed (https://www.ncbi.nlm.nih.gov/pubmed/), the common and unique mechanisms of the three classical prescriptions for the treatment of arrhythmia were compared from the extracted information.

### Experimental Validation

#### Experimental Model and Drug Treatment

Zhigancao Tang, GLT, and HET were purchased from the Granule Dispensing Department of Dongzhimen Hospital, Beijing University of Chinese Medicine. Calcium chloride was purchased from Xi'long Scientific Co., Ltd (Guangdong, China, Batch No: 200616-2).

The experimental procedures used in this study were approved by the Animal Welfare Ethics Committee of Sino Animal (Beijing) Science and Technology Development Co. Ltd. and were in accordance with the U.S. National Institutes of Health (NIH) (Publications No. 8023). A total of 48 8-week-old SD male rats were obtained from SPF Biotechnology Corporation Ltd. [experimental animal license No. SYXK (Beijing, China), 2020-0051]. The rats were placed in polycarbonate cages under a 12-h light/dark cycle in an air-conditioned room under a constant temperature (25 ± 1°C) and humidity (50 ± 10%) in a specific pathogen-free environment. They were randomly divided into the following five groups: control group (deionized water), vehicle group (deionized water), ZT group (ZT granule, 159.31 mg/100 g, 0.5 ml/100 g), GLT group (GLT granule, 16.72 mg/100 g, 0.5 ml/100 g), and HET group (HET granule, 91.12 mg/100 g, 0.5 ml/100 g). The rats were gavaged daily for 14 days and anesthetized 30 min after intragastric administration on the 14th day (6% chloral hydrate, 0.5 ml/100 g, intraperitoneally). Anesthetized rats were then linked to ECG and IV lead. ECGs were recorded using the PowerLab biological signal processing system. Once the ECG was stable, the control group was injected with saline (0.9% NaCl, 1.5 ml/min/kg) through the tail vein and the vehicle group, ZT, GLT, and HET groups were injected with calcium chloride solution (1.5 ml/min/kg, concentration 35 mg/ml) through the tail vein. The ECG was recorded for 15 min and the occurrence and duration time of ventricular arrhythmia (VA) were observed. The left cardiac myocardium (left ventricle) was frozen for analysis.

#### Quantitative Real-Time PCR (qRT-PCR)

Samples from five rats from each group were used for qRT-PCR. Total RNA was extracted from the left cardiac myocardium of each rat using TRIzol (Tiangen Biochemical Technology Corporation Ltd., Beijing, China) and reverse transcribed into complementary DNA (cDNA) using the Reverse Transcription System Kit (Takara, Shanghai, China) in accordance with the manufacturer's protocols. qRT-PCR was performed on the ABI 7500 Real-Time PCR System (Applied Biosystems, Foster City, California, USA) using the SYBR Green PCR Kit to determine messenger RNA (mRNA) expression levels. The relative expression of CALM1 (primers: GCTACATCAGTGCGGCAGA and ACCTGTCCGTCTCCATCAATA), CALM2 (primers: CGGGGATGGGACAATAACA and TACCGTCGGCATCTACTTCAT), CALM3 (primers: GGAATGG- CTACATCAGTGCTG and CCACTTCCTCATCAGTCAGCTT), CRP (primers: GGACAAATG- CAAGCATCATCT and GTGCCCGCCAGTTCAAA), interleukin-6 (IL-6) (primers: GATTGTATGAACAGCGATGATGC and AGAAACGGAACTCCAGAAGACC), and NOS3 (primers: GATCCTAACTTGCCTTGCATC and CTCAATGTCGTGTAATCGGTCT) was analyzed using the comparative CT method for relative quantitation and the 2-DDCt method by normalizing to β-actin expression and relative expression was presented as the percentage change compared to matched controls. All the quantitative data were obtained from at least three independent experiments and were presented as the mean ± SD. The SPSS statistical software version 27.0 (IBM Corporation, Armonk, New York, USA) was used for statistical analysis. The variances between the two groups were compared using Student's *t*-test and multiple groups were compared using the one-way ANOVA. *p* < 0.05 were considered as statistically significant.

## Results

### Related Ingredients and Targets of ZT, GLT, and HET

**Figure 1:**
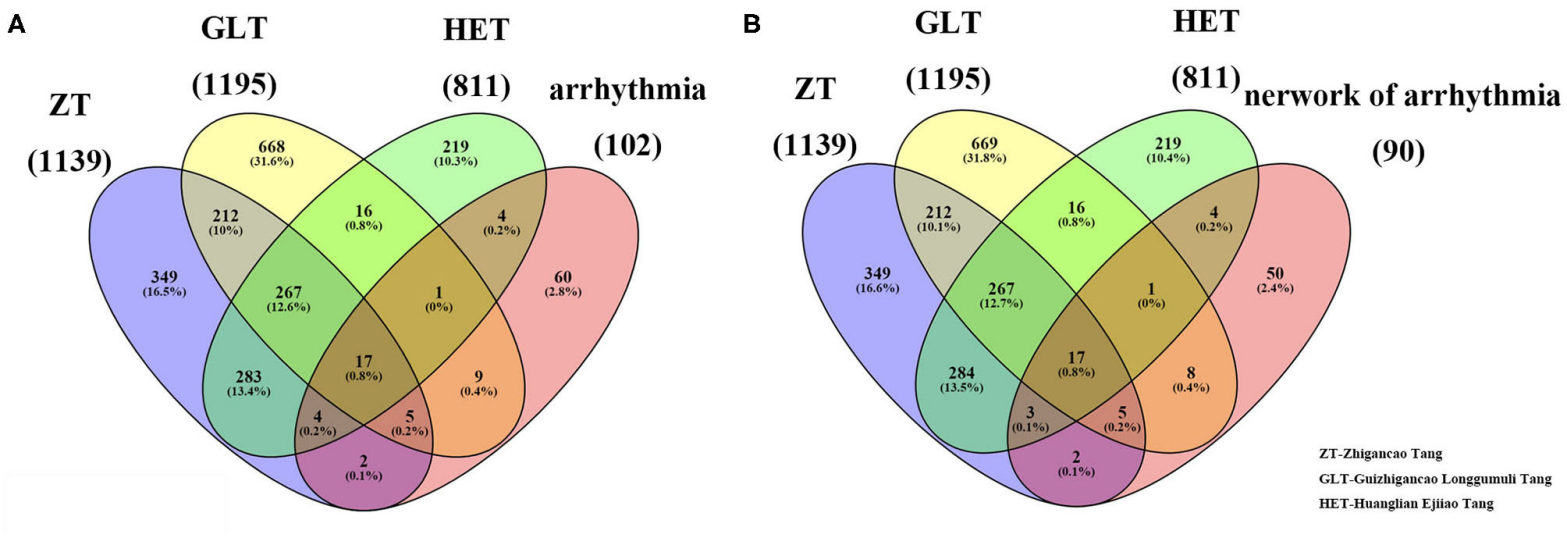
The related targets of the three fangjis and arrhythmia. **(A)** The overlapping targets among the three fangjis and arrhythmia; **(B)** The overlapping targets among the three fangjis and the network of arrhythmia.

Based on our search strategy involving the constituents of the three prescriptions, ZT (Glycyrrhizae Radix et Rhizoma, Zingiberis Rhizoma Recens, Ginseng Radix et Rhizoma, Rehmanniae Radix, Cinnamomi Ramulus, Asini Corii Colla, Ophiopogonis Radix, Cannabis Fructus, Vinum, and Jujubae Fructus), GLT (Cinnamomi Ramulus, Glycyrrhizae Radix et Rhizoma, Os Draconis, and Ostreae Concha), and HET (Scutellariae Radix, Coptidis Rhizoma, Paeoniae Radix Alba, Asini Corii Colla, and Gallus Gallus Domesticus), we retrieved 187, 105, and 68 active ingredients and 1,139, 1,195, and 811 corresponding gene targets for ZT, GLT, and HET, respectively (Supplementary Table 1). There were 351, 677, and 233 unique genes, respectively, associated with ZT, GLT, and HET, with 284 genes in common among the three decoctions (Figure 1A).

### Related Genes and Network Construction of Arrhythmia

**Figure 2:**
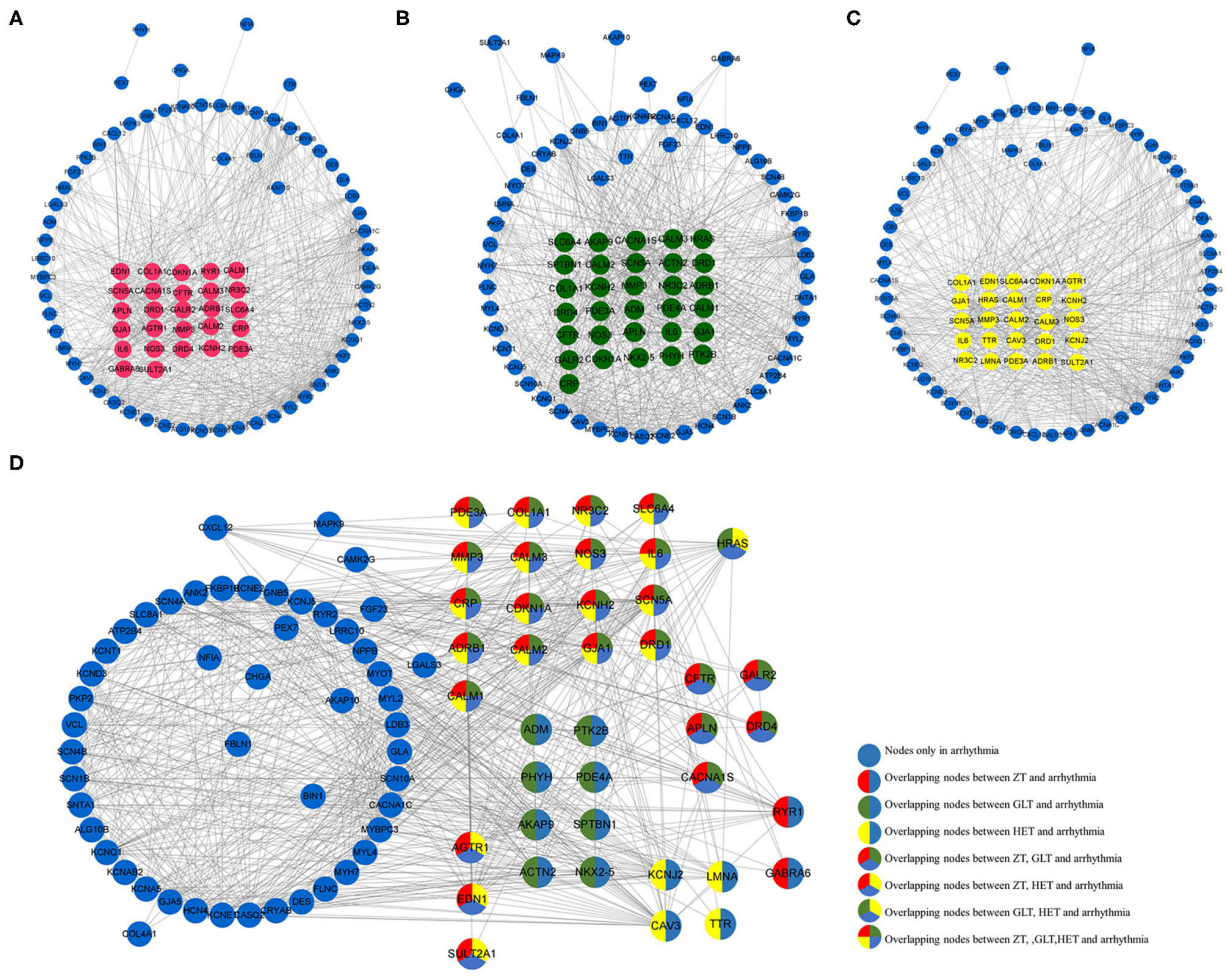
The distribution of the three fangjis in arrhythmia network. **(A–C)** marked with red, green, and yellow nodes that represent the targets of Zhigancao Tang (ZT), Guizhigancao Longgumuli Tang (GLT), and Huanglian E'jiao Tang (HET), respectively; **(D)** The overlapping and unique targets in the arrhythmia network.

**Figure 3:**
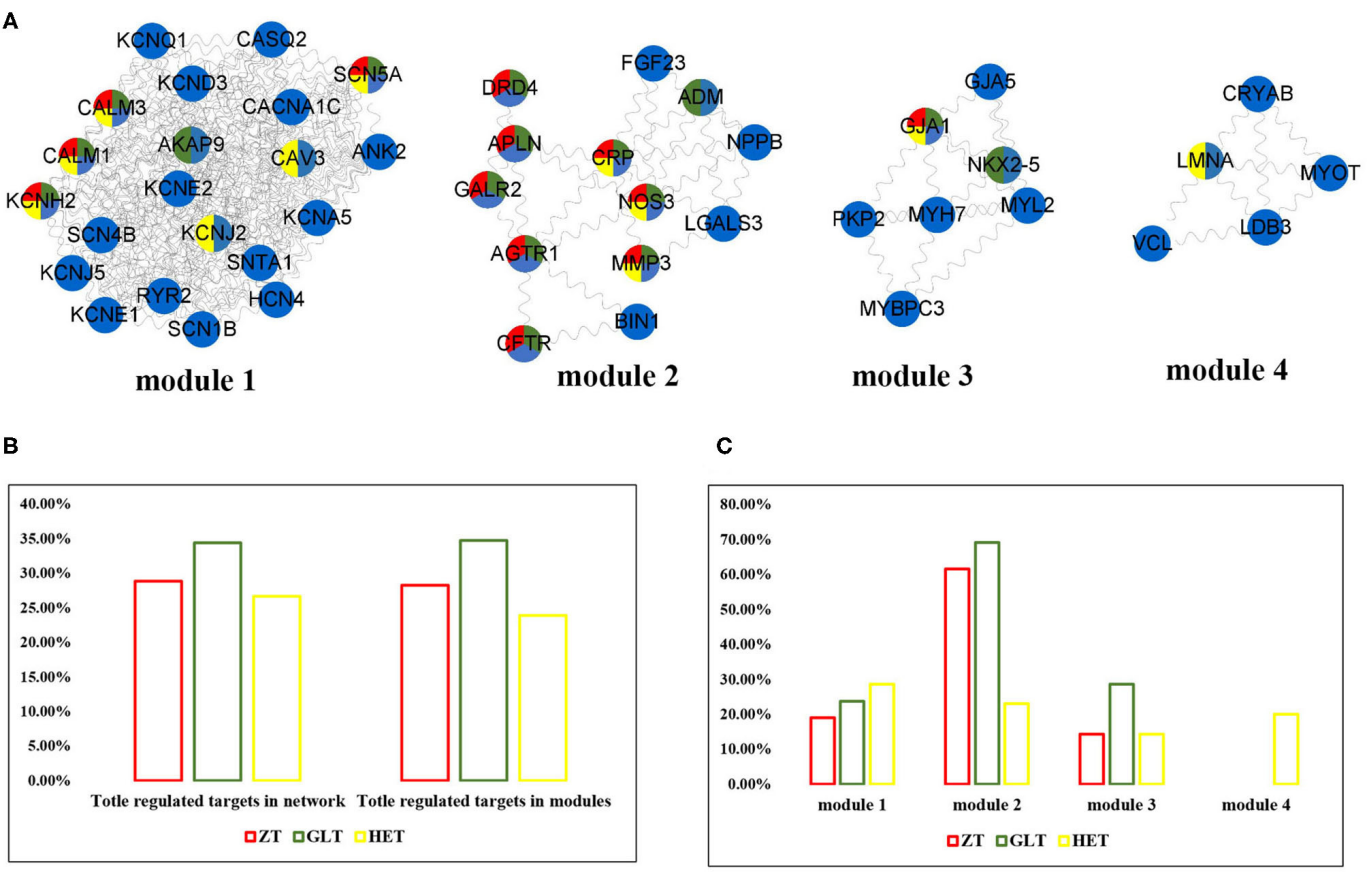
The distribution of three fangjis in arrhythmia modules. **(A)** The module division of arrhythmia network; **(B,C)** The coverage rate of three fangjis in arrhythmia network and modules.

Based on the NCBI database, 102 arrhythmia-related genes were obtained (Supplementary Table 2). After mapping to the STRING database, a network was constructed that included 90 nodes (88.2% of 102) and 666 edges. When the relevant targets of the three fangjis decoctions were compared with the arrhythmia-related genes, there were 42 overlapping genes. When the 90 node genes were compared with the arrhythmia-related targets, 40 (95.2% of 42) overlapping genes were assigned (Figure 1B), with the other two non-overlapping genes being *SLC22A5* and *ECHS1*. Analysis of the fangjis-related molecules in the arrhythmia network showed 27, 31, and 25 (30, 34.4, and 27.8% of 90) genes in the ZT, GLT, and HET decoctions, respectively, overlapped with the arrhythmia network (Figure 3B). In particular, 17 genes (*PDE3A, COL1A1, NR3C2, SLC6A4, MMP3, CALM3, NOS3, IL6, CRP, CDKN1A, KCNH2, SCN5A, ADRB1, CALM2, GJA1, DRD1*, and *CALM1*) were co-associated with ZT, GLT, HET, and arrhythmia; 5 genes (*CFTR, GALR2, APLN, DRD4, and CACNA1S*) were co-associated with ZT, GLT, and arrhythmia; 3 genes (*AGTR1, EDN1, and SULT2A1*) were co-associated with ZT, HET, and arrhythmia; and, lastly, 1 gene (*HRAS*) was co-associated with GLT, HET, and arrhythmia. There were 8, 4, and 2 unique genes for GLT, HET, and ZT, respectively, in the arrhythmia network (Figure 2).

### Diverse Distribution of the Three Decoctions in Arrhythmia Modules

As shown in Figure 3A, arrhythmia network was divided into 4 modules using the MCODE method. Among these prescriptions, 28.3, 34.8, and 23.9% of molecules for ZT, GLT, and HET were covered in the arrhythmia modules, respectively (Figure 3B). Modules 1, 2, and 3 were regulated by ZT, GLT, and HET simultaneously and module 4 was only regulated by HET (Figures 3A,C). According to the distribution of the fangjis-related genes, module two was considered to be the main regulator of ZT and GLT, covering 61.5 and 56.3% molecules, respectively. The HET-related molecular regulation was relatively scattered, covering 28.57, 23.08, 14.29, and 20% in modules 1 to 4, respectively.

### Functional Enrichment Analysis of the Three Fangjis Decoctions

**Figure 4:**
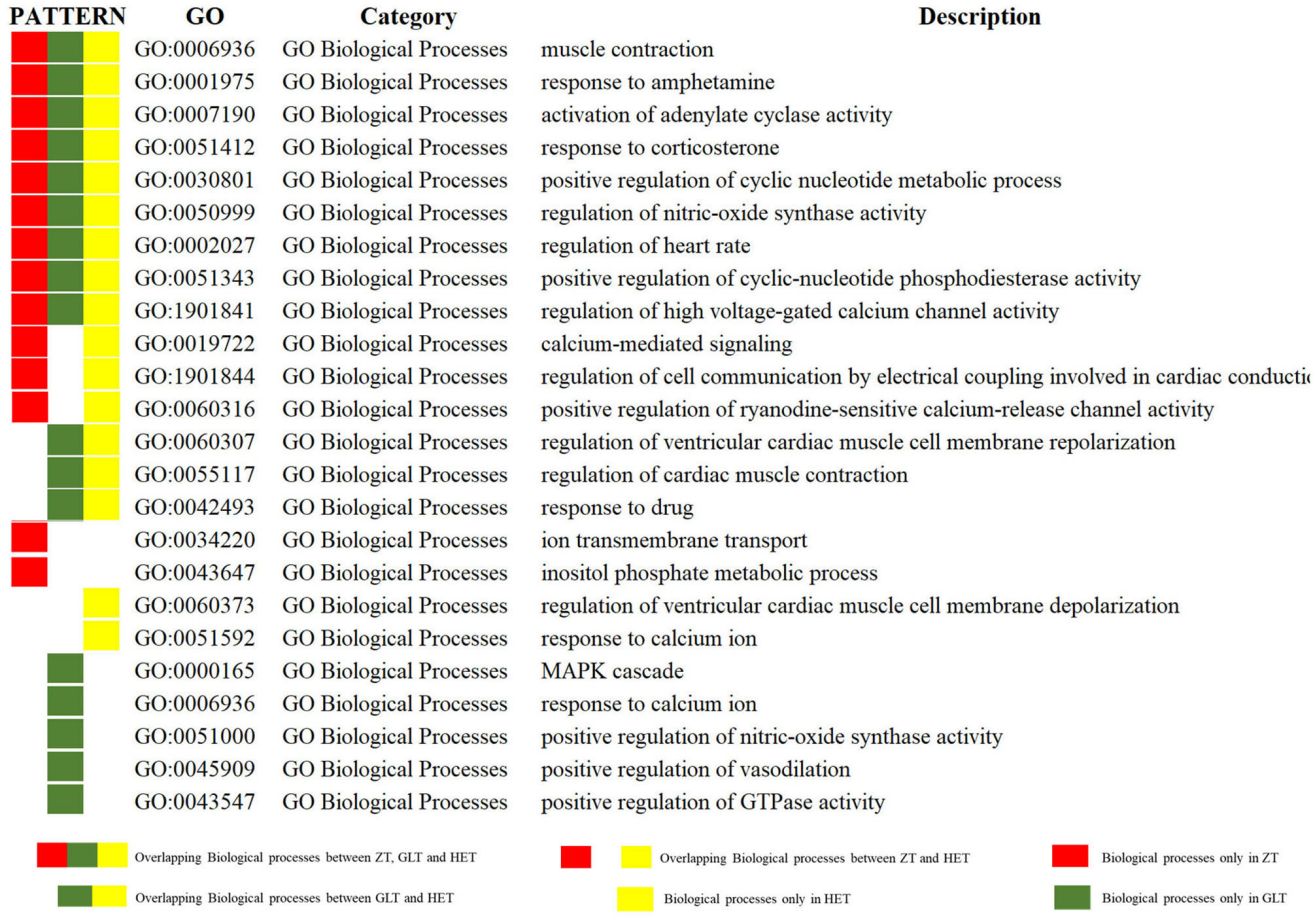
The Gene Ontology (GO) biological processes of ZT, GLT, and HET.

**Figure 5:**
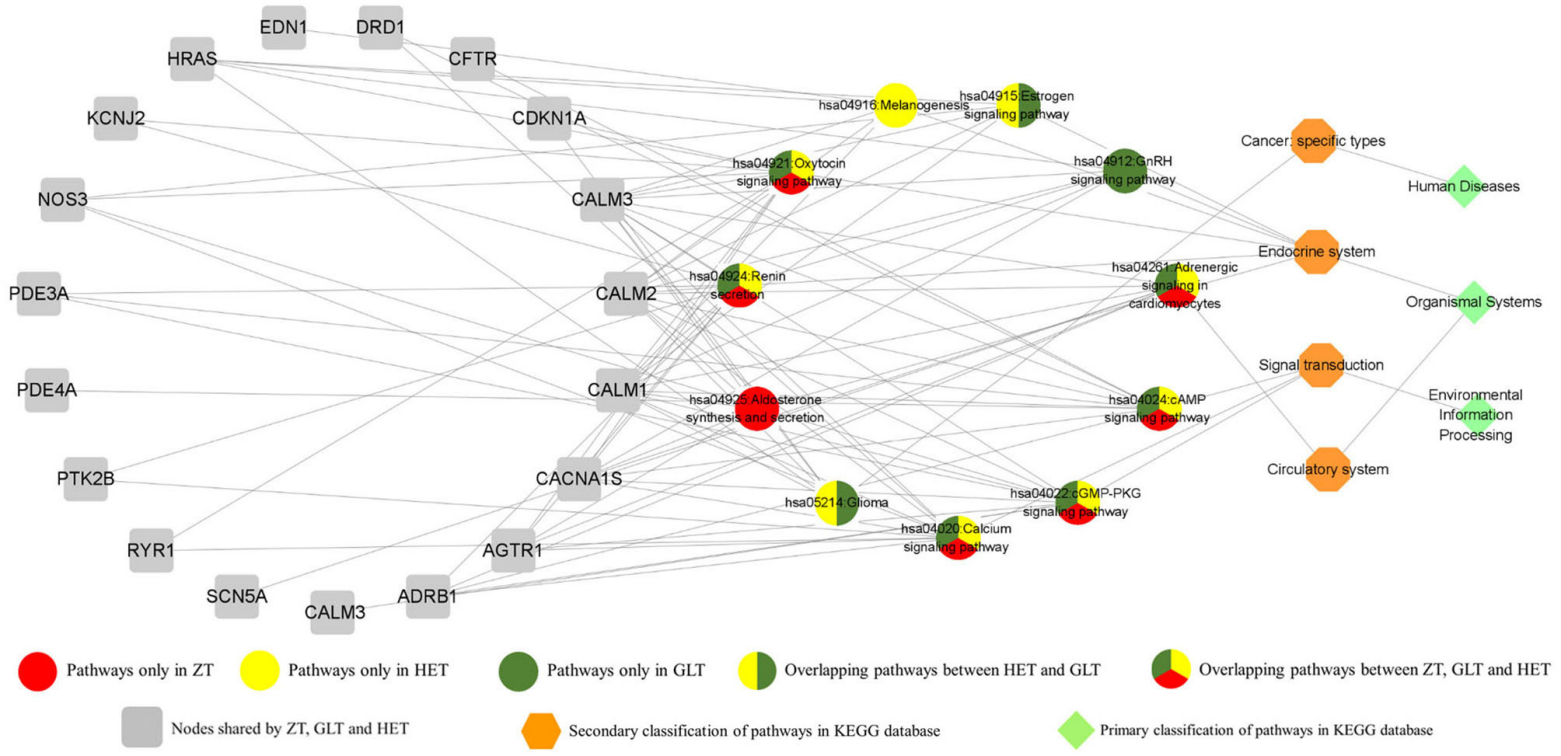
The pathways of ZT, GLT, and HET with its targets and the relationship.

The DAVID platform was used to identify significant enrichments in biological processes and signaling pathways. According to this analysis, a total of 14, 17, and 17 biological processes were associated with ZT, GLT, and HET, respectively (Supplementary Tables 3–5). Among these, 9 biological processes existed in common between ZT, GLT, and HET, 3 biological processes existed in common between ZT and HET, and 3 biological processes existed in common between GLT and HET. There were 2, 5, and 2 unique biological processes for ZT, GLT, and HET, respectively (Figure 4). With respect to signaling pathways, a total of 7, 9, and 9 significantly enriched pathways were identified in ZT, GLT, and HET, respectively (Supplementary Tables 6–8), of which 6 pathways were enriched in ZT, GLT, and HET, and 2 pathways were enriched in GLT and HET. Moreover, each of the three prescriptions was associated with a unique pathway, namely aldosterone synthesis and secretion for ZT, GnRH signaling pathway for GLT, and melanogenesis for HET (Figure 5).

### Effect of the Three Fangjis on ECG in Rats

**Figure 6:**
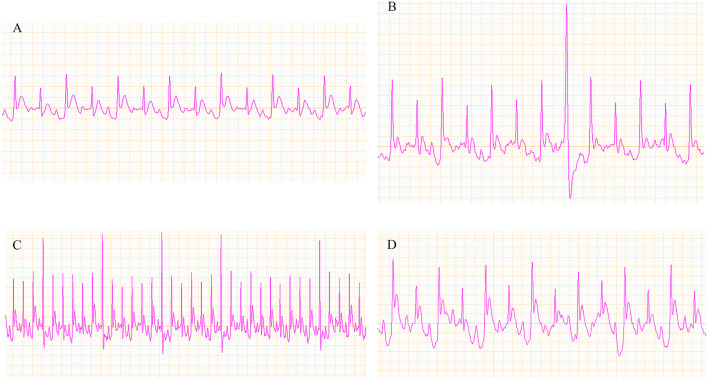
Representative ECGs of the control and vehicle groups. **(A)** ECG of the control group; **(B–D)** ECG of ventricular arrhythmia (VA).

**Figure 7:**
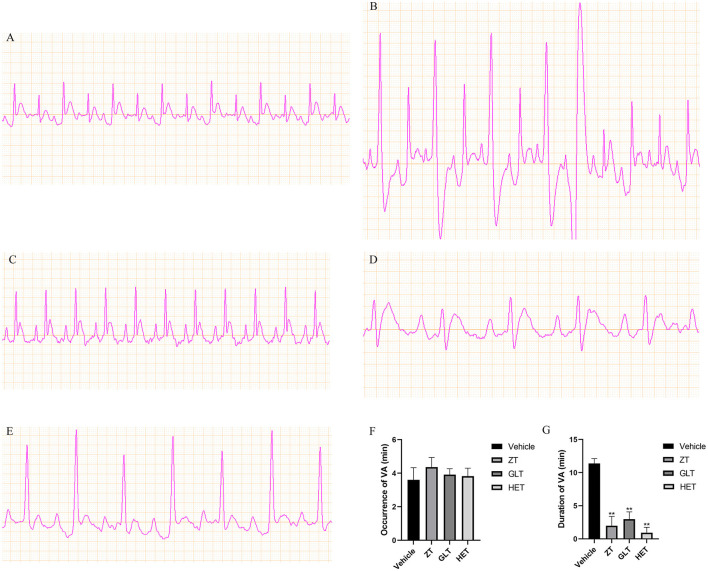
Representative ECGs of the different groups and the effect of the fangjis on ECG. **(A)** ECG of the control group; **(B)** ECG of the vehicle group; **(C)** ECG of the ZT group; **(D)** ECG of the GLT group; **(E)** ECG of the HET group; **(F)** Occurrence time of VA in rats with calcium chloride-induced VA; **(G)** Duration time of VA in rats with calcium chloride-induced VA.**p < 0.01.

Representative ECGs of the control and vehicle groups are shown in Figure 6 with the typical ECGs of the different treatment groups shown in Figure 7. This analysis shows that interventions with the different fangjis (ZT, GLT, and HET) had an effect on the occurrence and duration of VA. The typical ECGs of the different groups are shown in Figures 7A–E. ZT, GLT, and HET could prolong the occurrence of VA, but did not show statistically significant differences compared with the vehicle group. Furthermore, ZT, GLT, and HET significantly shortened the duration of VA (*p* < 0.01).

### Real-Time PCR

**Figure 8:**
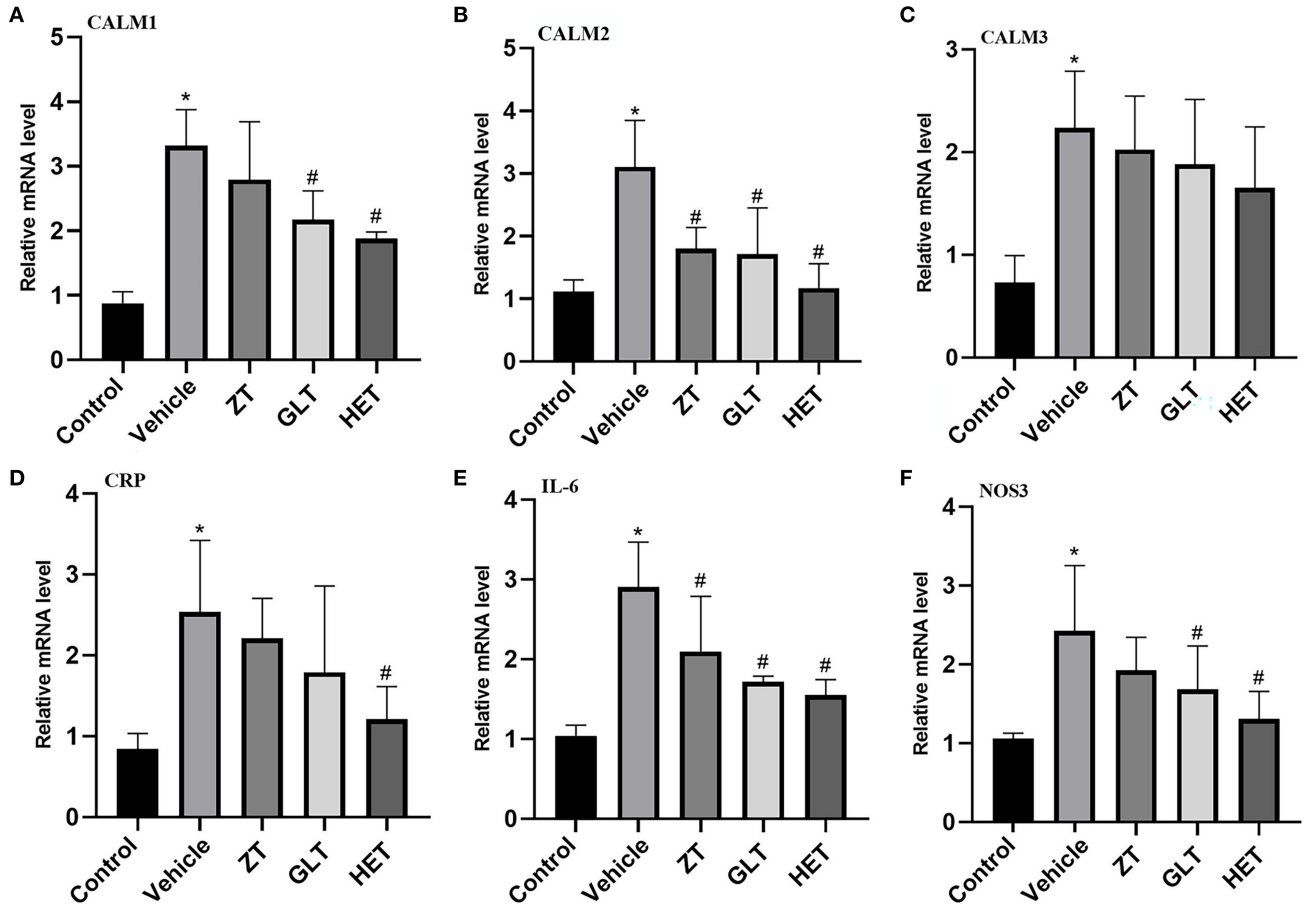
Experimental validation by real-time RT-PCR. *P < 0.05 **(A)** mRNA expression of CALM1; **(B)** mRNA expression of CALM2; **(C)** mRNA expression of CALM3; **(D)** mRNA expression of CRP; **(E)** mRNA expression of IL-6; **(F)** mRNA expression of NOS3.

The results of PCR analysis showed that the expression of CALM2 and IL-6 mRNAs were consistently altered in individual samples from the control, vehicle, ZT, GLT, and HET groups. CALM1 and NOS3 mRNA expression was altered in individual samples from the control, vehicle, GLT, and HET groups (Figures 8A–F). There were statistically significant differences between the vehicle and control groups (*p* < 0.05). The ZT, GLT, and HET groups showed statistically significant differences compared with the vehicle group (*p* < 0.05).

## Discussion

This study aimed to identify the similarities and characteristic therapeutic mechanisms of the different fangjis decoctions against arrhythmia using network- and module-based approaches. ZT, GLT, and HET have been used in clinical settings for a considerable period of time, playing an important role in complementary and alternative therapies. This method gathered potential targets for ZT, GLT, and HET to investigate the biological basis of treating arrhythmias and to clarify the precise clinical applications of the various prescriptions.

### Targeted Therapy of TCM Based on Biological Network Model

The etiology of complex diseases might be polygenic, perhaps even “omnigenic” (24–26). Therefore, network-based approaches represent powerful tools to help elucidate pathophysiological conditions including the occurrence and progression of arrhythmias. These methods emphasize the interactions from multiple dimensions involving molecules, pathways, cells, organs, and organisms (27) rather than the individual building blocks of complex systems (28). On this basis, the importance of multitarget combinational therapeutic strategies has become increasingly recognized (29). Fangji integrates the characteristics of multiple herbs, multibioactive components, multiple targets, and pathways (30), properties which are ideal for the treatment of complex disorders.

### Variation of Functional Effects Among the Three Fangjis Decoctions

By comparing the homogeneity and heterogeneity of the three prescriptions for arrhythmia, we found that: (1) although the molecules targeted by ZT, GLT, and HET are different, a majority of shared targets, biological processes, and pathways converge to denote certain primary characteristics; (2) the targeting rate of ZT, GLT, and HET in the network and modules was comparable, but the target distribution within the modules was different, for example, module two was the main target module for ZT and GLT, whereas module 1 was the main target for HET; (3) among the pathways enriched by the three prescriptions, most were related to the calcium signaling pathways; and (4) each fangjis were also uniquely associated with a particular target pathway.

Regardless of whether the target distribution was similar or identical, shared molecular backgrounds may target the arrhythmia network to produce pharmacological effects (31). This similarity may construct a drug prediction model via the interactions between chemical and genomic elements (32). Moreover, different combinations of functionally-related gene perturbations (mutations or gene expression changes) may be the cause of phenotypic differences (33) and the diversity of target distribution of these three prescriptions may be helpful in drug discovery. Gene clusters constructed by the network can enable us to comprehensively understand the molecular mechanisms behind complex diseases through pathway enrichment analysis (34). In addition, signaling pathways can further reflect the biological responses of cells to drugs (35). Under these circumstances, pathway analysis might contribute toward elucidating the mechanisms of reversing the arrhythmia pathologies, all of which were integrated into seven main categories based on the KEGG classification. Some core common and unique signaling pathways are listed below.

#### Shared Signaling Pathways of Pharmacogenetics

Based on the fangji-related target pathways network analysis, we found the commonly enriched pathways involving the three fangjis converged on signal transduction processed related to the cyclic adenosine 3′,5′-monophosphate (cAMP), cyclic guanosine 3′,5′-monophosphate (cGMP)-PKG, and calcium signaling pathways. Consistently, according to the Vaughan–Williams classification scheme, the effect on receptors and/or ion channels represents one of the primary mechanisms of antiarrhythmic drugs (36).

Cyclic adenosine 3′,5′-monophosphate is the one of the most important second messengers in the heart, which plays a critical role in cellular responses to extracellular stimuli in the cardiovascular system and regulates many physiological processes including heart contraction and relaxation (37, 38). The current study suggests that PDE4 controls excitation–contraction coupling (ECC) in the pig heart and ventricular myocytes, while PDE4 inhibitors exert inotropic and proarrhythmic effects upon PDE3 inhibition in the preclinical models (39). Interaction of PDE4 and EPAC2 is crucial for coordinating the proarrhythmic effect of cAMP (40). Moreover, cAMP can binds directly to the hyperpolarization-activated cyclic nucleotide gated (HCN) channel that is mainly expressed in heart node cells to increase pacemaker current (I_f_), thereby promoting an increase in heart rate (41).

Cyclic guanosine 3′,5′-monophosphate is a ubiquitous second messenger and its benefits are widely accepted. These chiefly involve the regulation of various physiological activities such as smooth muscle cell relaxation and heart contraction (38). The pathological role of cGMP signaling in cardiovascular disease has been mainly associated with hypertension (42), atherosclerosis (43), cardiac hypertrophy, and ventricular remodeling (44), etc., but notably it has rarely been implicated in arrhythmia.

Renin secretion and adrenergic signaling in cardiomyocytes are common pathways of the three fangjis decoctions. Physiologically, the fight-or-flight response is initiated by releasing norepinephrine (NE) from the cardiac sympathetic nerves and epinephrine (Epi) from the adrenal medulla, which binds to β-adrenergic receptors (β-ARs) on cardiomyocytes. This triggers a signaling cascade leading to an increase in cAMP and consequent protein kinase A (PKA) activation and phosphorylation of a myriad of targets, thereby increasing heart rate and conduction velocity, increasing contraction strength, and the speed of relaxation (45). However, excessive β-AR stimulation may cause electrophysiological abnormalities such as long QT syndrome (46) and even fatal heart rhythm disorders (47). These pathological mechanisms are based on the abnormality of ion channels (41). A meta-analysis suggested that renin-angiotensin system inhibitors may contribute to a reduction in the recurrence rate of atrial fibrillation after catheter ablation (48).

Interestingly, the estrogen (E2) signaling pathway was shared by GLT and HET, while the GnRH signaling pathway was only linked with GLT, although both the pathways belong to the same endocrine system category. Previous epidemiological studies suggested that women have a lower incidence of cardiovascular disease during their reproductive age, partly due to E2 (49). These further suggest potential gender differences in cardiac electrophysiology, which could affect the risk of arrhythmia and sudden cardiac death (49). These differences are probably regulated by sex hormones and these also affect the performance of arrhythmias and response to antiarrhythmic drugs and treatments (50). The current study on the role of E2 in reducing VA has been controversial. In a rat model of arrhythmia induced by coronary artery ligation, Philp et al. (51) reported dose-dependent effects of E2 in significantly reducing premature ventricular beats (PVBs) and ventricular fibrillation (VF) in female rats, although female E2 levels in myocardial ischemia and antiarrhythmic activity were larger than in male rats. This may be due to the effects of E2 on the expression and function of ion channels that control cardiac cell excitation and repolarization (52). However, some studies demonstrate that E2 actually exacerbates VAs. Yan et al. (53) showed that BPA combined with E2 increased the duration of VA following ischemia/reperfusion (I/R) injury in female rat hearts, possibly due to the altered calcium processing mediated by estrogen receptor β (ERβ) signaling in cardiomyocytes. However, Es also had a protective effect on infarction, underlying the cardioprotective effect of E2 in I/R injury (54). Notably, a randomized controlled trial indicating higher testosterone levels in men was associated with lower sudden cardiac arrest (SCA) events and higher E2 levels in women and men were associated with higher SCA events (55).

The aldosterone synthesis and secretion pathway were only shown in conjunction with ZT, but is related to renin secretion and adrenergic signaling in cardiomyocytes. A randomized, double-blind, placebo-controlled study showed that excess aldosterone secretion resulted in proarrhythmic effects and moreover confirmed that drugs affecting the renin-angiotensin-aldosterone system (RAAS) were of benefit for arrhythmia and its comorbidities (56). A systematic review provided evidence that ACEI/ARB and aldosterone inhibitors could prevent the recurrence of AF (57). Thus, RAAS inhibition has a tendency to be an upstream therapy for AF.

#### Core Functions of the Three Decoctions Focus on Calcium Signaling Pathway

**Figure 9:**
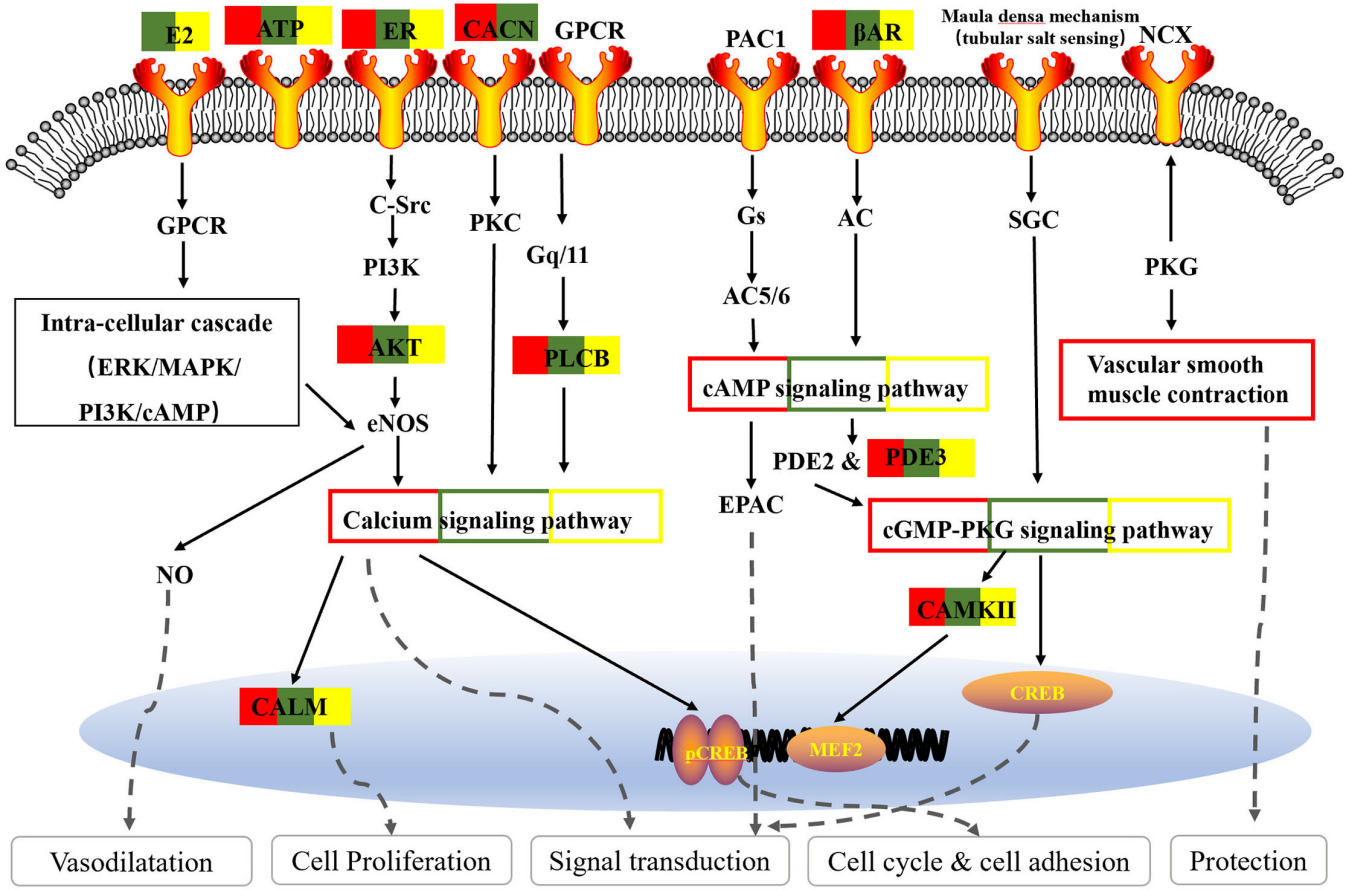
Schematic depiction target pathways identified in the three fangjis.

**Figure 10:**
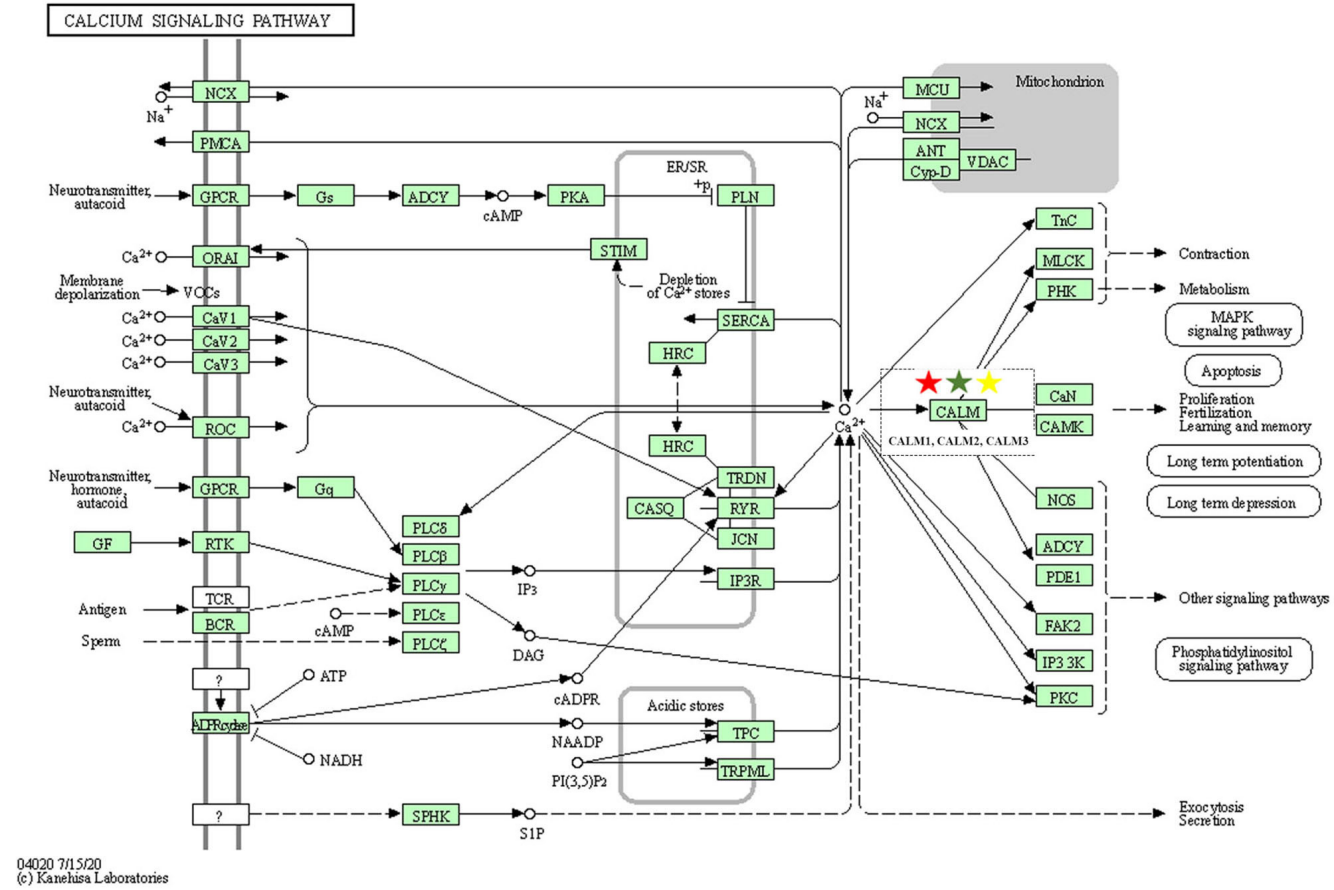
Calcium signaling pathway [the Kyoto Encyclipedia of Genes and Genomes (KEGG) database].

As one of the most common signal transduction molecules, the divalent cation calcium (Ca^2+^) can regulate a variety of biological functions including muscle contraction, cell exocytosis, neuronal activity, and also triggering of programmed cell death (58, 59). Notably, the majority of the pathway enrichments for all the fangjis decoctions were related to calcium signaling pathways such as the cAMP and cGMP-PKG pathways (Figure 9). The molecular mechanism of Ca^2+^ ions mediating various types of arrhythmias has been widely studied including congenital long QT syndrome (LQTS), catecholaminergic polymorphic ventricular tachycardia (CPVT), and atrial fibrillation (AF) (58). As shown in Figure 10, calmodulins (CaMs) are key checkpoint regulators in the calcium signaling pathway and inherited mutations in the CALM1; gene encoding CaM1 leads to the fourth subtype of CPVT-4 (60). Moreover, *CALM1* and *CALM2* genes have been linked to LQTS (61) and mutations in SCN5A-encoded Nav1.5 correlated with idiopathic ventricular fibrillation (IVF) (62) and Brugada syndrome (63). Besides these studies, there are limited reports associated with mutations in CALM3 with LQTS, except for one study showing elevated QTc intervals caused by D130G mutation in a neonate (64). Invasive studies in animals and humans have suggested that abnormal atrial Ca^2+^ signaling may play a role in pathophysiology of AF, leading to postpolarized triggering activity, conduction blocks, and Ca^2+^ driven subcellular alternation (65, 66). Indeed, the calcium signaling pathway is a pivotal nexus connecting the pathways associated with the activities of all the three fangjis prescriptions.

### Multiple Targeting Patterns of the Fangjis in the Network Promote Precision Medicine

Associations between phenotype and genotype have been extensively studied in the context of disease (67, 68). In theory, the association between genes and phenotypes is complex and dynamic due to different causal subtypes among the same disease. Therefore, multifunctional drugs or drug combinations have been proposed. A broad-spectrum target of TCM and the unique principle of “*Jun-Chen-Zuo-Shi*” (69, 70) provide a basis for prescription and pinpointing patients suitable for specific-targeted therapies. As an example, this study analyzed the three fangjis against arrhythmia based on networks and modules and compared the same and unique targeting modes, revealing various pharmacological mechanisms, providing effective and individualized treatment plans for patients.

## Conclusion

In summary, the pattern of “chemical composition-target-network” builds the association between the different fangjis and arrhythmia in complex systems, which provides a novel approach to differentiate the therapeutic mechanism of drugs. Drug targets can be traced clearly using network and module models and molecular distribution can be compared and discussed in multiple dimensions. This comprehensive approach defined the target spectrums of the three fangjis prescriptions from different angles including target, biological processes, and pathways. The identification of shared signaling pathways confirmed the significance of abnormal signal transduction underlying the pathological mechanisms driving arrhythmia, which were reflected in the targets of all three prescriptions. GLT and HET are particularly inclined toward conditioning abnormal hormone secretion, while ZT tended more toward effects on RAAS disorders. Importantly, calcium signaling pathways were identified as the intermediate regulator linking to multiple key signaling pathways associated with ion channels. This comparative pharmacological evidence may, therefore, help to select suitable decoction schedules for individualized interventions in arrhythmia.

## Data Availability Statement

The original contributions presented in the study are included in the article/Supplementary Material, further inquiries can be directed to the corresponding author.

## Ethics Statement

The animal study was reviewed and approved by the Animal Welfare Ethics Committee of Sino Animal (Beijing) Science and Technology Development Co.Ltd.

## Author Contributions

HL contributed to the conception or design of the study. XL and WX revised the manuscript. DL and YT are responsible for data collection. KY was responsible for statistics. PW drafted the manuscript. All authors read and approved the final version of the manuscript.

## Funding

This study was supported by Grants from the National Natural Science Foundation of China (No. 81273741) and China Postdoctoral Science Foundation (No. 2021M702311), and the National Administration of Traditional Chinese Medicine (No. 2019XZZX-XXG001).

## Conflict of Interest

The authors declare that the research was conducted in the absence of any commercial or financial relationships that could be construed as a potential conflict ofinterest.

## Publisher's Note

All claims expressed in this article are solely those of the authors and do not necessarily represent those of their affiliated organizations, or those of the publisher, the editors and the reviewers. Any product that may be evaluated in this article, or claim that may be made by its manufacturer, is not guaranteed or endorsed by the publisher.

## Abbreviations

PKG: protein kinase G
CALM2: calmodulin 2
NOS3: nitric oxide synthase 3
CRP: C-reactive protein
SD: standard deviation
NaCl: sodium chloride
2-DDCt: Dct Discrete Cosine Transform
SLC22A5: solute carrier family 22 member 5
ECHS1: enoyl-CoA hydratase shortchain 1
PDE3A: phosphodiesterase 3A
COL1A1: collagen type I alpha 1 chain
NR3C2: nuclear receptor subfamily 3 group C member 2
SLC6A4: solute carrier family 6 member 4
MMP3: matrix metallopeptidase 3
CDKN1A: cyclin dependent kinase inhibitor 1A
KCNH2: potassium voltage-gated channel subfamily H member 2
SCN5A: sodium voltage-gated channel alpha subunit 5
ADRB1: adrenoceptor beta 1
GJA1: gap junction protein alpha 1
DRD1: dopamine receptor D1
CFTR: CF transmembrane conductance regulator
GALR2: galanin receptor 2
APLN: apelin
DRD4: dopamine receptor D4
CACNA1S: calcium voltagegated channel subunit alpha1 S
AGTR1: angiotensin II receptor type 1
EDN1: endothelin 1
SULT2A1: sulfotransferase family 2A member 1
HRAS: HRas proto-oncogene
GTPase: GnRH: gonadotropin-releasing hormone
PDE4: phosphodiesterases 4
EPAC2: exchange protein directlyactivated by cAMP, also now as
RAPGEF4: Rap guanine nucleotide exchange factor 4
BPA: bisphenol
ACEI: angiotensin-converting enzyme inhibitors
ARB: angiotensin receptor blockers
CaM1: calmodulin 1
CPVT-4: catecholaminergic polymorphic ventricular tachycardia-4
SCN5A: sodium voltage-gated channel alpha subunit 5.
