# Supplementary material for: Integrated Pharmacogenetics Analysis of the Three Fangjis Decoctions for Treating Arrhythmias Based on Molecular Network Patterns
Source: Front Cardiovasc Med. 2021 Dec 24;8:726694. doi: 10.3389/fcvm.2021.726694 (PMC8739471; doi:10.3389/fcvm.2021.726694)
Supplement: Supplementary file 1 [file Data_Sheet_1.docx]

**Supplementary table 1 Active ingredients and corresponding targets of three formulas**

| Formulas | Herbs | Active Ingredients | Target proteins |
| --- | --- | --- | --- |
| Zhigancao Tang  (ZT) | Gancao Dazao Renshen Shengdihuang Maren Maidong Guizhi Shengjiang Baijiu Ejiao | quercetin  mairin  jaranol isorhamnetin sitosterol formononetin calycosin kaempferol licochalcone a vestitol inermine dfv glycyrol medicarpin lupiwighteone 7-methoxy-2-methyl isoflavone naringenin (2s)-2-[4-hydroxy-3-(3-methylbut-2-enyl)phenyl]-8,8-dimethyl-2,3-dihydropyrano[2,3-f]chromen-4-one euchrenone glyasperin b glyasperin f glyasperin c isotrifoliol (e)-1-(2,4-dihydroxyphenyl)-3-(2,2-dimethylchromen-6-yl)prop-2-en-1-one kanzonols w (2s)-6-(2,4-dihydroxyphenyl)-2-(2-hydroxypropan-2-yl)-4-methoxy-2,3-dihydrofuro[3,2-g]chromen-7-one semilicoisoflavone b glepidotin a glepidotin b phaseolinisoflavan glypallichalcone 8-(6-hydroxy-2-benzofuranyl)-2,2-dimethyl-5-chromenol licochalcone b licochalcone g 3-(2,4-dihydroxyphenyl)-8-(1,1-dimethylprop-2-enyl)-7-hydroxy-5-methoxy-coumarin licoricone gancaonin a gancaonin b licorice glycoside e 3-(3,4-dihydroxyphenyl)-5,7-dihydroxy-8-(3-methylbut-2-enyl)chromone 5,7-dihydroxy-3-(4-methoxyphenyl)-8-(3-methylbut-2-enyl)chromone 2-(3,4-dihydroxyphenyl)-5,7-dihydroxy-6-(3-methylbut-2-enyl)chromone glycyrin licocoumarone licoisoflavone licoisoflavone b licoisoflavanone shinpterocarpin (e)-3-[3,4-dihydroxy-5-(3-methylbut-2-enyl)phenyl]-1-(2,4-dihydroxyphenyl)prop-2-en-1-one liquiritin licopyranocoumarin 3,22-dihydroxy-11-oxo-delta(12)-oleanene-27-alpha-methoxycarbonyl-29-oic acid glyzaglabrin glabridin glabranin glabrene glabrone 1,3-dihydroxy-9-methoxy-6-benzofurano[3,2-c]chromenone 1,3-dihydroxy-8,9-dimethoxy-6-benzofurano[3,2-c]chromenone eurycarpin a glycyroside (-)-medicocarpin sigmoidin-b (2r)-7-hydroxy-2-(4-hydroxyphenyl)chroman-4-one (2s)-7-hydroxy-2-(4-hydroxyphenyl)-8-(3-methylbut-2-enyl)chroman-4-one isoglycyrol isolicoflavonol hmo 1-methoxyphaseollidin quercetin der. 3'-hydroxy-4'-o-methylglabridin 3'-methoxyglabridin 2-[(3r)-8,8-dimethyl-3,4-dihydro-2h-pyrano[6,5-f]chromen-3-yl]-5-methoxyphenol inflacoumarin a icos-5-enoic acid kanzonol f 6-prenylated eriodictyol 7,2',4'-trihydroxy－5-methoxy-3－arylcoumarin 7-acetoxy-2-methylisoflavone 8-prenylated eriodictyol gadelaidic acid gancaonin g gancaonin h licoagrocarpin glyasperins m glycyrrhiza flavonol a licoagroisoflavone 18α-hydroxyglycyrrhetic acid odoratin phaseol xambioona dehydroglyasperins c beta-sitosterol stigmasterol fumarine diop inermin chrysanthemaxanthin aposiopolamine celabenzine deoxyharringtonine dianthramine arachidonate frutinone a ginsenoside rh2 ginsenoside-rh4_qt girinimbin gomisin b malkangunin panaxadiol suchilactone alexandrin_qt ginsenoside rg5_qt (-)-catechin (+)-catechin stepholidine protoporphyrin berberine (s)-coclaurine beta-carotene ziziphin_qt ruvoside_qt nuciferin 21302-79-4 moupinamide stepharine spiradine a zizyphus saponin i_qt jujuboside a_qt coumestrol daechuine s6 daechuine s7 jujubasaponin v_qt jujuboside c_qt mauritine d (3s,6r,8s,9s,10r,13r,14s,17r)-17-[(1r,4r)-4-ethyl-1,5-dimethylhexyl]-10,13-dimethyl-2,3,6,7,8,9,11,12,14,15,16,17-dodecahydro-1h-cyclopenta[a]phenanthrene-3,6-diol gamma-aminobutyric acid catalpol campesterol acteoside rehmaglutin a rehmaglutin b rehmaglutin c rehmaglutin d glutinoside luteolin (z)-3-(4-hydroxy-3-methoxy-phenyl)-n-[2-(4-hydroxyphenyl)ethyl]acrylamide arachidonic acid gondoic acid arginine histidine lysine uridine guanosine diosgenin orchinol ruscogenin n-trans-feruloyltyramine ophiopogonanone e 6-aldehydo-isoophipogonone a 6-aldehydo-isoophipogonone b methyl ophiopogonanone a methyl ophiopogonanone b ophiopogon b ophiopogonanone a ophiopogonone a ophiopogonanone c ophiopogonin c ophiopogon a ophiopogonone b ophiopogonin b ophiopogonin a ophiopogonin d ent-epicatechin (-)-taxifolin taxifolin peroxyergosterol 6-methylgingediacetate2 poriferast-5-en-3beta-ol dihydrocapsaicin ethyl aldehyde 17beta-oestradiol amyl butyrate amyl acetate ethyl caffeate glycerol sinapate malvidin-3-arabinoside | PTGS1 AR PPARG PTGS2 HSP90AA1 NCOA2 DPP4 AKR1B1 PRSS1 TOP2B SERPIND1 KCNH2 SCN5A F7 ADRB2 MMP3 RXRA ACHE GABRA1 MAOB RELA EGFR AKT1 VEGFA CCND1 BCL2 BCL2L1 FOS CDKN1A EIF6 BAX CASP9 PLAU MMP2 MMP9 MAPK1 IL10 EGF RB1 CD40LG JUN IL6 AHSA1 CASP3 TP53 ELK1 NFKBIA POR ODC1 XDH CASP8 TOP1 SOD1 PRKCA MMP1 HIF1A STAT1 RUNX1T1 CDK1 HSPA5 ERBB2 ACACA HMOX1 CYP3A4 CYP1A2 CAV1 MYC F3 GJA1 CYP1A1 ICAM1 IL1B CCL2 SELE VCAM1 PTGER3 CXCL8 PRKCB BIRC5 DUOX2 NOS3 HSPB1 TGFB1 SULT1E1 MGAM IL2 NR1I2 CYP1B1 CCNB1 PLAT THBD SERPINE1 COL1A1 IFNG ALOX5 IL1A MPO TOP2A NCF1 ABCG2 HAS2 GSTP1 NFE2L2 NQO1 PARP1 AHR PSMD3 SLC2A4 COL3A1 CXCL11 CXCL2 DCAF5 NR1I3 CHEK2 INSR CLDN4 PPARA PPARD HSF1 CRP CXCL10 CHUK SPP1 RUNX2 RASSF1 E2F1 E2F2 ACPP CTSD IGFBP3 IGF2 IRF1 ERBB3 PON1 DIO1 PCOLCE NPEPPS HK2 NKX3-1 RASA1 GSTM1 GSTM2 PGR NOS2 ESR2 CDK2 CHEK1 PCP4 ESR1 MAPK14 GSK3B CCNA2 PYGM NCOA1 GRIA2 OLR1 NR3C2 CHRM1 PDE3A ADRA1D SLC6A3 SLC6A4 PKIA LACTBL1 IL4 SIRT1 ATP5B MT-ND6 SLC6A2 CHRM2 ADRA1B IKBKB MAPK8 AKR1C3 SLPI CA2 STAT3 CDK4 FOSL2 CHRM4 HTR2A CHRM3 HTR3A OPRM1 KDR DRD1 CHRM5 MAPK10 OPRD1 ADRB1 LTA4H MAPK3 FASN LDLR BAD CAT MTTP APOB PLB1 HMGCR CYP19A1 UGT1A8 SREBF1 GSR ABCC1 ADIPOQ SOAT2 AKR1C1 GOT1 ABAT CES1 SOAT1 ITM2C RXRB CHRNA2 MAP2 ADRA2A MAOA CTRB1 CACNA1S RXRG CASP1 ADCYAP1 PSMG1 MAP2K4 NR3C1 KLF7 ADH1C DRD5 ADRA2C DRD4 HTR2C ADRA2B PDE10A ALB CTNNB1 CASP7 MMP10 DRD2 CYP2B6 CDC37 MDM2 APBA3 PCNA MCL1 TYR XIAP PTGES NUF2 ADCY2 MET ENSG00000196689 G6PD TNFRSF1A PECAM1 PLA2G4A SELP GLB1 ALDH2 ABCA1 ALDH3A1 UCP2 C1R CETP ABCG1 ABCC4 KCNK10 TNFRSF1B PTGES2 KCNK2 COL1A2 AASS ABCB1 ABCC11 ABCG5 ABCG8 ABL1 ACE ACSS1 ACTC1 ACVR2B ADCY1 ADCY3 ADCY4 ADCY5 ADCY6 ADCY7 ADCY9 ADORA1 ADPRH ADRA1A AGPAT3 AGT AGTR1 AGTR2 AICDA AIMP1 AIMP2 AK1 AKR1A1 ALAD ALDH1A1 ALDH1B1 ALDH3A2 ALDH3B2 ALDH5A1 ALDH7A1 ALDH9A1 ALKBH3 ALPPL2 ANKRA2 ANXA1 AOX1 APLN APLNR APOA1 APOE APP AQP4 AQP5 AQP9 ARAF AREG ARG1 ARG2 ART1 ART3 ART5 ASL ASS1 ATP5A1 ATP5C1 AVP AVPR1A AZI2 BDKRB1 BDKRB2 BDNF BIRC3 BLMH BMP1 BMP2 BMP4 BMP6 BTD C3 C3AR1 C5 CA1 CA12 CA3 CALCA CALR CAPN1 CAPN9 CASP2 CASP6 CCKAR CCKBR CCL19 CCL20 CCL21 CCL25 CCL28 CCND2 CCNE1 CCR1 CCR2 CCR5 CCR6 CCR7 CCR8 CD22 CD36 CD40 CDA CDC14B CDC25A CDH1 CDH2 CDK6 CDKN1B CEBPB CELA1 CETN2 CGA CKB CNDP1 CNDP2 CNR1 COMT CPB2 CPLX1 CPS1 CREB1 CRH CRHBP CSF2 CSN1S1 CTGF CTSS CUL1 CXCL1 CXCL13 CXCL16 CXCL3 CXCL5 CXCL6 CXCL9 CXCR1 CXCR2 CXCR5 CXCR6 CYP17A1 CYP2C19 CYP2C8 CYP2C9 CYP2E1 CYP3A5 CYP3A7 CYP7A1 CYSLTR1 CYSLTR2 DARS DCN DCUN1D1 DDAH1 DDC DECR1 DEFB4A DEK DHCR24 DLG1 DLG3 DNMT1 DNMT3A DRD3 EDN1 EDN2 EDN3 EDNRA EEF1E1 EGR1 EGR3 ELN ENAH EPO ERAP1 ERBB4 F2 F2R F2RL1 F2RL2 F2RL3 FABP1 FABP2 FANCD2 FAS FASLG FCGRT FES FFAR1 FFAR2 FGF2 FGF7 FGFR2 FLT1 FN1 FNTA FPR1 FPR2 FSHB FSHR FZD2 GABARAPL1 GABBR2 GABRA2 GABRA3 GABRA4 GABRA6 GABRB1 GABRB3 GABRG1 GABRG2 GABRG3 GABRP GABRR1 GABRR2 GAD1 GAD2 GAL GALR1 GALR2 GALR3 GAMT GAP43 GAPDH GATM GCG GCH1 GCLC GGCT GHR GHRH GHSR GJB1 GLRX GLUL GNA14 GNA15 GNAI1 GNAQ GNAT2 GNB2 GNB3 GNG12 GNG3 GNG4 GNG5 GNG7 GNGT1 GNGT2 GNMT GNRH1 GNRH2 GNRHR GPR156 GPR17 GPR18 GPR55 GPR65 GPRC6A GPX4 GRAP2 GRB14 GRB2 GRIA1 GRK2 GRM1 GRN GRP GRPR GSTM3 GSTM5 GUCY1A3 GUCY1B3 GZMB GZMH H2AFZ HAL HAMP HARS2 HBEGF HBZ HCRTR1 HCRTR2 HDAC8 HDC HEBP1 HGFAC HMGCS2 HOXB13 HPGD HPN HPRT1 HPSE HRH3 HRH4 HSD11B1 HSD17B12 HSD17B2 HSD17B3 HSD17B6 HSD17B7 HSPA4 HSPA8 HTR1A HTR1B HTR1D HTR1E HTR1F HTR2B HTR5A ID1 IGF1 IGF1R IGFBP2 IGFBP4 IGFBP5 IHH IL11 IL1RN IL4I1 IL5 IMPDH2 IRF4 IRS1 ITGA4 KARS KAT5 KCNJ3 KIFAP3 KISS1 KISS1R KLF9 KLK5 KLK8 KNG1 LARS LCMT2 LEP LEPR LHB LNPEP LPAR2 LPAR3 LPAR4 LPAR6 LPL LRP6 LYZ MAP2K1 MARS MASP1 MCHR1 MCHR2 MDH2 METTL2B MFGE8 MLNR MMP13 MMP7 MSN MTNR1A MTNR1B MVK MYOD1 NAGS NCK2 NFYA NGF NMBR NMU NMUR1 NMUR2 NOTCH1 NPFF NPFFR1 NPFFR2 NPM1 NPPA NPY NPY1R NPY5R NR1H2 NRF1 NT5C NT5C1A NT5C2 NT5E NTS NTSR1 NTSR2 OAT OPN4 OPRK1 OTC OVGP1 OXT OXTR P2RY1 P2RY10 P2RY12 P2RY13 P2RY14 P2RY2 P2RY6 PADI4 PAQR7 PDIA3 PDYN PDZK1 PENK PER2 PFN1 PIK3CA PIK3R1 PIK3R2 PIP PITX1 PLCB1 PLCB2 PLCB3 PLCB4 PLCG1 PLK1 PNKP PNMT PNOC POMC PPBP PPP5C PRKCZ PRL PRLR PROK1 PROK2 PROKR1 PROKR2 PSMA2 PSMA3 PSMA4 PSMA5 PSMA6 PSMB1 PSMB2 PSMB4 PSMB5 PSMB6 PSMB7 PSMC1 PSMC2 PSMC3 PSMC4 PSMC5 PSMC6 PSMD10 PSMD11 PSMD14 PSMD4 PSMD5 PSMD7 PSME1 PSME2 PSME3 PSME4 PTAFR PTGDS PTGER1 PTGFR PTGIS PTHLH PTK2 PTP4A1 PTTG1 PTX3 PYY QARS RAB3A RABEP1 RAC1 RAC2 RAF1 RARS RARS2 RBM5 RBP4 REN RETN RGS1 RGS10 RGS14 RGS19 RGS2 RGS20 RGS4 RHO RHOU RNASE1 RPS6KB1 RXFP4 RPN2 S100A11 S100A7 S1PR1 S1PR4 S1PR5 SCG2 SCNN1G SEC61A1 SEC61B SEC61G SERPINB1 SERPINC1 SERPINH1 SETD7 SHBG SHH SIRT5 SIRT6 SKP2 SLC12A2 SLC15A3 SLC15A4 SLC16A10 SLC17A7 SLC22A1 SLC22A11 SLC22A2 SLC22A3 SLC22A8 SLC28A1 SLC28A2 SLC29A1 SLC29A2 SLC2A2 SLC32A1 SLC38A4 SLC38A5 SLC3A1 SLC6A1 SLC6A11 SLC6A12 SLC6A13 SLC7A1 SLC7A3 SLC7A4 SLC7A5 SLC7A6 SLC7A7 SLC7A9 SLCO1A2 SLCO1B3 SLCO2B1 SLCO4A1 SMAD3 SMARCA4 SOCS2 SOST SST SSTR1 SSTR2 SSTR4 SSTR5 ST8SIA1 STAT5A STAT5B STC2 STIP1 STYK1 SYK SYT1 TAC1 TAC3 TACR2 TACR3 TERT TFF1 TGFA TGFBR2 TH TIMP2 TJP1 TLL1 TNFRSF11B TPH1 TPP2 TRAF2 TRH TRHR TRPA1 TRPM8 TSG101 TYMP TYRP1 UCK1 UCK2 UGT2A3 UGT2B10 UGT2B7 UPRT UTS2R VIP VLDLR WNT10B WNT2 XCL1 XCL2 XCR1 ZHX2 ZMYM6 ADH1A ADH4 ADH5 ADORA3 ALDH1A3 ALKBH2 ARHGEF25 ATP2A1 AVPR1B BACE1 C5AR1 CCK CCL16 CCL27 CCL5 CCR10 CCR4 CNR2 DDAH2 DEFA1 DIO3 DLK1 DMD DNASE1 EMP2 FNTB FPR3 GABBR1 GABRA5 GABRD GABRQ GAST GCGR GNG10 GNG2 GNRHR2 GPR132 GPX3 GREB1 GRIN2B GRK5 GZMM HCAR2 HLA-C HMGB1 HP HSD3B1 HSD3B2 IARS INS IRS2 ITGB3 LCK LCMT1 LTB4R MDM4 MME MT-CO1 MT-CO2 MTOR NF1 NMB NPB NPBWR1 NPBWR2 NPS NPSR1 NPW NPY2R NPY4R NT5C1B NT5M OCLN OPRL1 OSTN P2RY4 PARVB PIK3R3 PMCH PNP PPIA PSMB10 PSMB11 PSMB3 PSMD12 PSMD13 PTGDR2 PTPN1 QRFP RXFP3 RYR1 S100A10 S100A3 S1PR3 SLC22A4 SLC28A3 SLC29A3 SLC6A14 SOCS3 SRC SREBF2 SSTR3 SULT1A1 TCL1B TGM2 TNF UCKL1 UGT1A10 UGT1A3 UGT1A4 UGT1A7 UGT1A9 UGT2B15 UGT2B17 UPP1 VAMP2 WDR5 WT1 ZDHHC17 PPY GIP GNAI2 GNAI3 ZNF32 DNAJC5 NCL GABRB2 ADCY8 C7 GPR26 LPAR1 RGS13 RGS18 GABRE MIR224 TYRL ACAT1 ORC2 SLC25A2 RPS27A UBB UBC UBA52 HLA-A HLA-B ORC1 SLC25A15 GNA11 EPRS ART4 DOK1 SPR TACR1 LAT2 SLC7A8 UCN2 UTS2 HCRT PPOX PSMD2 RPN1 GPR4 GPR19 C6 PSMA7 BAP1 MAGI1 TF H3F3A H3F3B RAC3 CKM SCD SLC22A5 HIST1H4H HIST1H4A HIST1H4B HIST1H4C HIST1H4D HIST1H4E HIST1H4F HIST1H4I HIST1H4J HIST1H4K HIST1H4L HIST2H4A HIST4H4 NT5C3A POMP ADH1B ADH6 AHRR NCOA3 TRAM1 HGF HTT UGT1A1 UGT1A5 SULT2A1 ST2 UGT1A6 UGT2B4 UGT2B11 AMH MIF HBA1 HBA2 FOLH1 FOLH1B SERPINB10 SERPINB2 HBB HBD HBG1 HBG2 TPSAB1 TPSB2 HLA-DQB1 HLA-DRB1 DDR2 TKT DTL TNFSF13B BPNT1 PTEN TEP1 PTENP1 SLCO1B1 LST1 CLGN SCARB1 CFTR CPA6 CYP2A6 CALM3 CALM1 CALM2 L1CAM CABP1 S100G ANG RNASE4 CD79A CHAT PASK STK39 SCARB2 SMO SMOX CAD CALD1 EIF3A NOTCH3 NOTCH4 FOXM1 MPP2 CXCR4 LAP3 |
| Guizhigancao longgumuli Tang(GLT) | Guizhi Gancao Longgu Muli | (-)-taxifolin beta-sitosterol sitosterol (+)-catechin ent-epicatechin taxifolin peroxyergosterol inermine dfv mairin glycyrol jaranol medicarpin isorhamnetin lupiwighteone 7-methoxy-2-methyl isoflavone formononetin calycosin kaempferol naringenin (2s)-2-[4-hydroxy-3-(3-methylbut-2-enyl)phenyl]-8,8-dimethyl-2,3-dihydropyrano[2,3-f]chromen-4-one euchrenone glyasperin b glyasperin f glyasperin c isotrifoliol (e)-1-(2,4-dihydroxyphenyl)-3-(2,2-dimethylchromen-6-yl)prop-2-en-1-one kanzonols w (2s)-6-(2,4-dihydroxyphenyl)-2-(2-hydroxypropan-2-yl)-4-methoxy-2,3-dihydrofuro[3,2-g]chromen-7-one semilicoisoflavone b glepidotin a glepidotin b phaseolinisoflavan glypallichalcone 8-(6-hydroxy-2-benzofuranyl)-2,2-dimethyl-5-chromenol licochalcone b licochalcone g 3-(2,4-dihydroxyphenyl)-8-(1,1-dimethylprop-2-enyl)-7-hydroxy-5-methoxy-coumarin licoricone gancaonin a gancaonin b licorice glycoside e 3-(3,4-dihydroxyphenyl)-5,7-dihydroxy-8-(3-methylbut-2-enyl)chromone 5,7-dihydroxy-3-(4-methoxyphenyl)-8-(3-methylbut-2-enyl)chromone 2-(3,4-dihydroxyphenyl)-5,7-dihydroxy-6-(3-methylbut-2-enyl)chromone glycyrin licocoumarone licoisoflavone licoisoflavone b licoisoflavanone shinpterocarpin (e)-3-[3,4-dihydroxy-5-(3-methylbut-2-enyl)phenyl]-1-(2,4-dihydroxyphenyl)prop-2-en-1-one liquiritin licopyranocoumarin 3,22-dihydroxy-11-oxo-delta(12)-oleanene-27-alpha-methoxycarbonyl-29-oic acid glyzaglabrin glabridin glabranin glabrene glabrone 1,3-dihydroxy-9-methoxy-6-benzofurano[3,2-c]chromenone 1,3-dihydroxy-8,9-dimethoxy-6-benzofurano[3,2-c]chromenone eurycarpin a glycyroside (-)-medicocarpin sigmoidin-b (2r)-7-hydroxy-2-(4-hydroxyphenyl)chroman-4-one (2s)-7-hydroxy-2-(4-hydroxyphenyl)-8-(3-methylbut-2-enyl)chroman-4-one isoglycyrol isolicoflavonol hmo 1-methoxyphaseollidin quercetin der. 3'-hydroxy-4'-o-methylglabridin licochalcone a 3'-methoxyglabridin 2-[(3r)-8,8-dimethyl-3,4-dihydro-2h-pyrano[6,5-f]chromen-3-yl]-5-methoxyphenol inflacoumarin a icos-5-enoic acid kanzonol f 6-prenylated eriodictyol 7,2',4'-trihydroxy－5-methoxy-3－arylcoumarin 7-acetoxy-2-methylisoflavone 8-prenylated eriodictyol gadelaidic acid vestitol gancaonin g gancaonin h licoagrocarpin glyasperins m glycyrrhiza flavonol a licoagroisoflavone 18α-hydroxyglycyrrhetic acid odoratin phaseol xambioona dehydroglyasperins c quercetin calcium carbonate calcium phosphate magnesium ferric oxide calcium sulphate aluminum silicon | PTGS1 ESR1 PTGS2 HSP90AA1 LACTBL1 PGR NCOA2 KCNH2 DRD1 CHRM3 CHRM1 SCN5A CHRM4 PDE3A HTR2A ADRA1D CHRM2 ADRA1B ADRB2 CHRNA2 SLC6A4 OPRM1 GABRA1 BCL2 BAX CASP9 JUN CASP3 CASP8 PRKCA TGFB1 PON1 MAP2 NR3C2 PCP4 RXRA CAT HAS2 APOB AR PPARG DPP4 AKR1B1 PRSS1 TOP2B SERPIND1 F7 MMP3 ACHE MAOB RELA EGFR AKT1 VEGFA CCND1 BCL2L1 FOS CDKN1A EIF6 PLAU MMP2 MMP9 MAPK1 IL10 EGF RB1 CD40LG IL6 AHSA1 TP53 ELK1 NFKBIA POR ODC1 XDH TOP1 SOD1 MMP1 HIF1A STAT1 RUNX1T1 CDK1 HSPA5 ERBB2 ACACA HMOX1 CYP3A4 CYP1A2 CAV1 MYC F3 GJA1 CYP1A1 ICAM1 IL1B CCL2 SELE VCAM1 PTGER3 CXCL8 PRKCB BIRC5 DUOX2 NOS3 HSPB1 SULT1E1 MGAM IL2 NR1I2 CYP1B1 CCNB1 PLAT THBD SERPINE1 COL1A1 IFNG ALOX5 IL1A MPO TOP2A NCF1 ABCG2 GSTP1 NFE2L2 NQO1 PARP1 AHR PSMD3 SLC2A4 COL3A1 CXCL11 CXCL2 DCAF5 NR1I3 CHEK2 INSR CLDN4 PPARA PPARD HSF1 CRP CXCL10 CHUK SPP1 RUNX2 RASSF1 E2F1 E2F2 ACPP CTSD IGFBP3 IGF2 IRF1 ERBB3 DIO1 PCOLCE NPEPPS HK2 NKX3-1 RASA1 GSTM1 GSTM2 NOS2 ESR2 CDK2 CHEK1 MAPK14 GSK3B CCNA2 PYGM NCOA1 GRIA2 OLR1 SLC6A3 PKIA IL4 SIRT1 ATP5B MT-ND6 SLC6A2 IKBKB MAPK8 AKR1C3 SLPI CA2 STAT3 CDK4 FOSL2 HTR3A KDR CHRM5 MAPK10 OPRD1 ADRB1 LTA4H MAPK3 FASN LDLR BAD MTTP PLB1 HMGCR CYP19A1 UGT1A8 SREBF1 GSR ABCC1 ADIPOQ SOAT2 AKR1C1 GOT1 ABAT CES1 SOAT1 ITM2C RXRB AASDHPPT AASS ABCB10 ABCB6 ABL1 ACLY ACP5 ACSL1 ACTC1 ACTR3 ADAM17 ADAMTS1 ADCY1 ADCY2 ADCY3 ADCY4 ADCY5 ADCY6 ADCY7 ADCY9 ADI1 ADK ADM ADM2 ADORA1 ADORA2B ADPRH ADPRHL2 AGT AGTR2 AKAP9 ALAD ALDH2 ALPL ALPP ALPPL2 ANTXR2 ANXA1 AP2B1 APLN APLNR APP ARF1 ARF4 ARF6 ARFIP2 ARHGAP1 ARHGAP20 ARHGAP35 ARHGDIA ARHGDIB ARHGEF1 ARHGEF11 ARL1 ARL2BP ARL3 ARL6 ARL8A ARL8B ARPC1B ARPC2 ARPC3 ARSA ASAP3 ASS1 ATP1A1 ATP1B1 ATP2C2 ATP5A1 ATP5C1 ATP5D ATP5F1 ATP5J ATPIF1 AURKA AVP AVPR2 B4GALT1 BCHE BCKDHB BCKDK BDKRB1 BDKRB2 BDNF BOC BPHL BST2 BUB1 C1S C3 C3AR1 C5 CA6 CACNA1S CALB1 CALCA CALCB CALCR CALM3 CAMK2A CAMK2D CASC3 CASP1 CASP6 CBL CCL19 CCL20 CCL21 CCL25 CCL28 CCNT1 CCR1 CCR5 CCR6 CCR7 CCR8 CD207 CD209 CDC42 CDIPT CDK9 CDKN3 CDS1 CENPE CERK CHKA CHPT1 CKB CKMT2 CLDN16 CLEC7A CNDP2 CNR1 COL1A2 COL2A1 COMT COPG1 COPZ1 COX5A COX5B COX6A2 COX6B1 COX7A1 COX7C CPS1 CREB1 CRH CSF2 CSK CSN1S1 CSNK1G2 CSNK1G3 CSNK2A1 CTDSP1 CXCL1 CXCL13 CXCL16 CXCL3 CXCL5 CXCL6 CXCL9 CXCR1 CXCR2 CXCR5 CXCR6 CYB5B CYC1 CYTH2 DCK DCLRE1B DCTD DDX10 DDX18 DDX19B DDX58 DHH DIAPH1 DICER1 DIRAS1 DIRAS2 DLAT DLD DLG4 DNAJC6 DNM1 DNTT DOCK9 DRD2 DRD3 DRD4 DRD5 DTYMK DUT ECH1 ECHS1 EEA1 EHD2 EIF4A3 ENDOG ENO1 ENO2 ENO3 ENOSF1 ERBIN ERI1 ERI3 ERN1 ESD ETNK1 ETNK2 EXOC2 EXOC8 EXOG EXOSC10 EYA2 F10 F11R F2 F9 FABP2 FADS2 FAH FAHD1 FARS2 FBP1 FDFT1 FDPS FDX1 FEN1 FGA FGB FGFR2 FGG FLAD1 FMO2 FMR1 FNTA FOXA3 FOXP2 FOXP3 FPR1 FPR2 FSHR FTH1 FTMT FXN GAK GAL GALK1 GALR1 GALR2 GALR3 GBP1 GCC2 GCG GCLC GCLM GEM GGA1 GGPS1 GHRH GHRHR GIMAP2 GIMAP4 GIP GLP1R GLP2R GLUL GNA12 GNA13 GNAI1 GNAL GNAO1 GNAQ GNAS GNAT1 GNAZ GNB2 GNB3 GNB4 GNG11 GNG12 GNG13 GNG3 GNG4 GNG5 GNG7 GPR17 GPR18 GPR55 GRB2 GRHPR GRIN2C GRIN2D GRK2 GRM1 GRM5 GSG2 GSN GSS GUCY1A3 GUCY1B3 GYG1 HACL1 HEBP1 HMGCL HPGDS HPRT1 HRH2 HRH3 HRH4 HSPA2 HSPA8 HSPD1 HTR1A HTR1B HTR1D HTR1E HTR1F HTR4 HTR5A HTR6 HTR7 ICAM3 ICAM5 IDI1 IDI2 IGF1R IL18 ILF2 ILK IMPA1 INPP1 INPP4A INPP4B INPP5A INPP5D INPPL1 IPO13 ITGA10 ITGA11 ITGA2 ITGA2B ITGA4 ITGA5 ITGAD ITGAL ITGAX ITGB2 ITGB7 ITPA ITPKB JAK2 KIF11 KIF18A KIF1A KIF22 KIF23 KIF2C KIF3B KIF3C KIF5B KIF7 KIF9 KIFC3 KNG1 KPNB1 KRAS KRIT1 KSR2 LAMA1 LAMB1 LAP3 LHCGR LIG3 LIPT1 LPAR2 LPAR3 LY96 MAD2L1 MAGOH MAGT1 MALT1 MAP2K1 MAP2K2 MAP2K4 MAPK13 MAPKAPK2 MAPKAPK3 MARCO MC3R MC4R MC5R MCHR1 MCHR2 ME2 MERTK MET MLPH MRAS MSH2 MSH6 MST1R MTHFS MTNR1A MTNR1B MVD NADSYN1 NAE1 NAMPT NAPEPLD NCBP1 NCBP2 NCF2 NDC80 NDRG2 NEDD8 NEK2 NEK7 NFATC2 NGF NGFR NMNAT2 NMNAT3 NMRK1 NMU NMUR1 NMUR2 NPY NPY1R NPY5R NT5C NT5C1A NT5C2 NUDT5 NUF2 NUP153 NUTF2 OMG OPRK1 OTOF P2RY12 P2RY13 P2RY14 PACSIN3 PAK4 PAPSS1 PARD6B PASK PCNA PDE11A PDE1A PDE1B PDE3B PDE4A PDE4C PDE5A PDE6D PDE7B PDE8A PDE8B PDE9A PDHB PDK1 PDK3 PDK4 PDLIM4 PDXK PDYN PENK PFKFB3 PFKFB4 PGK1 PGM2 PHYH PI4K2A PI4K2B PIGR PIK3C2A PIK3C2B PIK3CA PIK3CB PIK3R1 PIK3R2 PIM1 PIP5K1A PIP5K1B PKM PLAA PLCB2 PLCB3 PLCE1 PLCG1 PLK1 PLXNA2 PLXNB1 PMM1 PNKP PNMT PNOC POLB POLH POLL POLM POMC PPA1 PPBP PPIP5K2 PPM1B PPM1K PPM1L PPP2R4 PRDX1 PRKAA1 PRKAB2 PRKACA PRKACB PRKACG PRKAG1 PRKAR2A PRKAR2B PROCR PRPS1 PSAT1 PSIP1 PSMA2 PSMA3 PSMA4 PSMA5 PSMA6 PSMB1 PSMB2 PSMB4 PSMB5 PSMB6 PSMB7 PTGDR PTGER2 PTGIR PTH PTH1R PTH2 PTH2R PTHLH PTK2 PTK2B PTPRB PTS PYY QPRT RAB11A RAB11FIP2 RAB14 RAB18 RAB1A RAB21 RAB22A RAB23 RAB25 RAB26 RAB27A RAB27B RAB28 RAB2A RAB30 RAB31 RAB33B RAB3A RAB3D RAB4A RAB4B RAB5A RAB6A RAB6B RAB7A RAB8A RAB9A RAB9B RABEP1 RABGEF1 RAC1 RAC2 RAF1 RALA RALB RALBP1 RALGDS RANBP1 RAP2A RBKS RECQL REM1 REN RERG REV1 RGS1 RGS10 RGS16 RGS2 RGS4 RGS8 RGS9 RHEB RHOQ RHOU RILP RNASE1 RNGTT ROCK1 RP2 RPIA RRAGC RRAGD RRAS RRAS2 RRM1 RTN4 RXFP1 RXFP2 RXFP4 S1PR1 S1PR4 S1PR5 SAE1 SARS SEPHS1 2-Sep SETD7 SETMAR SFN SGK1 SLC19A2 SLC19A3 SLC34A2 SLC41A1 SLC41A2 SMPD2 SMPD3 SNAP25 SNRPA SNUPN SPAG9 SPTBN1 SQLE SRM SST SSTR1 SSTR2 SSTR4 SSTR5 STX1A SYNJ2 TAAR1 TALDO1 TAOK2 TBX2 TBX5 TDG TERF2 TGM3 TKT TLR4 TNFAIP3 TNFRSF1A TNFSF15 TNK2 TNNC2 TNNI2 TPX2 TRAT1 TRPC3 TRPC6 TRPM6 TRPM7 TSHR TTL TUBB2B TUBG1 TUFM TYMS UBA3 UBASH3A UBE2A UBE2M UBLCP1 UCHL3 UCHL5 UCK2 UFC1 UNC119 UPF1 UPF3B UQCR11 UQCRB UQCRC1 UQCRC2 UQCRH UQCRQ UTRN VCP VIP VIPR1 VIPR2 VPS4B VPS54 VRK2 WARS WEE1 WRN XPO5 XRCC1 YWHAG YWHAZ ZAP70 ZBTB16 ZC3H12A ZNF24 ACSM2A ADORA2A ADORA3 ADRA2B ADRA2C ADRB3 ADSSL1 ALKBH2 APRT ARHGEF25 ARL2 ATP2A1 ATP5O BCKDHA BMPR2 C5AR1 CCL16 CCL27 CCL5 CCR10 CCR4 CD47 CDC42BPB CFD CHM CIB1 CNR2 DAPK1 DDX39B DDX52 DNASE1 DPP3 FNTB FOXO4 FPR3 GCGR GIMAP1 GNG10 GNG2 GP1BB GPX1 GRIN2A GRIN2B GRK1 GRM7 HCAR2 HSPA1B INPP5B INPP5F INPP5J ITGA1 ITGB3 LRRK2 MAPK11 MAPK12 MC2R MDM4 MT-CO1 MT-CO2 MT-CO3 MT-CYB MYL6B MYO6 NF1 NKX2-5 NPB NPBWR1 NPBWR2 NPS NPSR1 NPW NPY2R NPY4R NRAS NT5M NUDT3 OPRL1 P2RY4 PARVA PDE2A PDE4B PDXP PHF2 PIP5K1C PKLR PLA2G6 PLCG2 PMCH PNP POFUT2 PPIA PSAP PSMB3 PTGDR2 PTPN1 RAB11B RAB11FIP3 RAB12 RASSF5 RBM8A RELN RPE RXFP3 S1PR3 SCT SMYD3 SRC SSTR3 THTPA TMEM173 TPK1 TRPV5 TUBB3 UPP1 UQCR10 VAMP2 RND1 GNAI3 PRKAR1A PRKAR1B RPS27A UBB UBC UBA52 RHOA HRAS NUDT10 NUDT9 PSPH PSPHP1 ADCY8 CALM1 CALM2 TF LAMB2 LAMC1 RAC3 RHOD RHO BPNT1 PIP GNAI2 RND3 CFTR DTL TNFSF13B GNE NM GCK GK PDHX PDX1 PTEN TEP1 PTENP1 RNASEH1 RNH1 APEX1 HAP1 BRCA2 FANCB HSPA6 HSPA7 CHAT CHKB ADCYAP1R1 SCTR PPY EIF2AK1 EIF2AK2 DAP IAPP POLK POLQ NT5C3A POMP ARX UBA2 CASP10 PPIG MOCOS MOS MCS PMS2 PMS2P1 DNPEP KCNK4 PAK1 PAK2 ADRA2A ZNF32 RANBP2 RGPD5 RGPD8 RHOC KIT RAD1 RRAD TK1 TK2 ANTXR1 ATR PTGER4 FAS C7 KLK6 KLK7 DPYSL2 DRP2 RAB1B OSR1 OXSR1 ISY1 FOXK1 FOXK2 MIP TNPO1 EPHA3 FOXM1 MPP2 PDPK1 EPHB4 GBP3 ATL1 GPR26 LPAR1 PAK3 ACTN2 ACTN3 CD1A CD1D OCRL CTDSP2 SCP2 FBP2 KHSRP CHD2 DSCAM HGF SOS1 KIF2A APOBEC3F APOBEC3G MAL MKL1 EPHB2 CAD DFFB CD79B ILF3 MPP4 C6 PSMA7 ASMT ATP5G1 ATP5G2 GARS GART TUBB2A TUBB KLK1 HK1 |
| Huangliang E’jiao Tang(HET) | Huangqin Huanglian Baishao Ejiao Jizihuang | quercetin magnograndiolide palmidin a palmatine berberine coptisine worenine berberrubine epiberberine (r)-canadine berlambine corchoroside a_qt moupinamide obacunone ent-epicatechin wogonin (2r)-7-hydroxy-5-methoxy-2-phenylchroman-4-one beta-sitosterol sitosterol stigmasterol norwogonin 5,2'-dihydroxy-6,7,8-trimethoxyflavone bis[(2s)-2-ethylhexyl] benzene-1,2-dicarboxylate supraene acacetin baicalein diop 5,8,2'-trihydroxy-7-methoxyflavone 5,7,2,5-tetrahydroxy-8,6-dimethoxyflavone carthamidin 2,6,2',4'-tetrahydroxy-6'-methoxychaleone dihydrobaicalin_qt eriodyctiol (flavanone) salvigenin 5,2',6'-trihydroxy-7,8-dimethoxyflavone 5,7,2',6'-tetrahydroxyflavone dihydrooroxylin a skullcapflavone ii oroxylin a panicolin 5,7,4'-trihydroxy-8-methoxyflavone neobaicalein dihydrooroxylin moslosooflavone 11,13-eicosadienoic acid, methyl ester 5,7,4'-trihydroxy-6-methoxyflavanone 5,7,4'-trihydroxy-8-methoxyflavanone rivularin mairin kaempferol (+)-catechin 11alpha,12alpha-epoxy-3beta-23-dihydroxy-30-norolean-20-en-28,12beta-olide paeoniflorgenone (3s,5r,8r,9r,10s,14s)-3,17-dihydroxy-4,4,8,10,14-pentamethyl-2,3,5,6,7,9-hexahydro-1h-cyclopenta[a]phenanthrene-15,16-dione lactiflorin paeoniflorin paeoniflorin_qt albiflorin_qt benzoyl paeoniflorin arginine histidine lysine cholesterol oleinic acid linoleic acid lutein linolenic acid lutein-3-linolenate | PTGS1 AR PPARG PTGS2 HSP90AA1 NCOA2 DPP4 AKR1B1 PRSS1 TOP2B SERPIND1 KCNH2 SCN5A F7 ADRB2 MMP3 RXRA ACHE GABRA1 MAOB RELA EGFR AKT1 VEGFA CCND1 BCL2 BCL2L1 FOS CDKN1A EIF6 BAX CASP9 PLAU MMP2 MMP9 MAPK1 IL10 EGF RB1 CD40LG JUN IL6 AHSA1 CASP3 TP53 ELK1 NFKBIA POR ODC1 XDH CASP8 TOP1 SOD1 PRKCA MMP1 HIF1A STAT1 RUNX1T1 CDK1 HSPA5 ERBB2 ACACA HMOX1 CYP3A4 CYP1A2 CAV1 MYC F3 GJA1 CYP1A1 ICAM1 IL1B CCL2 SELE VCAM1 PTGER3 CXCL8 PRKCB BIRC5 DUOX2 NOS3 HSPB1 TGFB1 SULT1E1 MGAM IL2 NR1I2 CYP1B1 CCNB1 PLAT THBD SERPINE1 COL1A1 IFNG ALOX5 IL1A MPO TOP2A NCF1 ABCG2 HAS2 GSTP1 NFE2L2 NQO1 PARP1 AHR PSMD3 SLC2A4 COL3A1 CXCL11 CXCL2 DCAF5 NR1I3 CHEK2 INSR CLDN4 PPARA PPARD HSF1 CRP CXCL10 CHUK SPP1 RUNX2 RASSF1 E2F1 E2F2 ACPP CTSD IGFBP3 IGF2 IRF1 ERBB3 PON1 DIO1 PCOLCE NPEPPS HK2 NKX3-1 RASA1 GSTM1 GSTM2 GRIA2 NOS2 ESR1 ESR2 PCP4 CDK2 PDE10A CHEK1 CHRM3 CHRM1 CHRM5 HTR3A ADRA2C CHRM4 OPRD1 HTR2A HTR2C ADRA1B SLC6A3 ADRA1D SLC6A4 OPRM1 DRD1 DRD5 SLC6A2 CHRM2 NR3C2 LACTBL1 PDE3A MAPK14 GSK3B KDR BBC3 TEP1 PRKCD FN1 MCL1 PKIA CHRNA2 MAP2 PGR ADH1C NCOA1 ADRA2A LTA4H MAOA CTRB1 ADRB1 FASN FASLG CYP19A1 FOSL1 FOSL2 CYCS ALOX12 NFATC1 TDRD7 EGLN1 NOX5 FABP5 APOD PYGM CDK7 CYP2C9 CA2 IKBKB MAPK8 AKR1C3 SLPI CAT CD14 LBP AASS ABCA1 ABCA10 ABCA3 ABCB1 ABCB4 ABCG1 ABCG4 ABCG5 ABCG8 ACADM ACE ACOT2 ACOT4 ACSBG2 ACSL1 ACSL3 ACSL4 ADAM10 ADPRH ADRA1A AGT AGTR1 AIMP1 AIMP2 ALAS1 ALB ALOX15 ALOX15B ANKRD1 ANXA1 ANXA7 APOA1 APOA2 APOA4 APOA5 APOB APOC3 APOE APOF APP ARG1 ARG2 ARNTL ART1 ART3 ART5 ASL ASS1 ATF3 ATF4 ATP1A1 ATP1B1 AVP AVPR1A AZI2 BCO1 BDKRB1 BDKRB2 BLMH BTD CA1 CALM3 CALR CARM1 CAV3 CCKAR CCKBR CCR2 CD36 CDC42 CDK19 CDK8 CELA1 CETP CH25H CNDP1 CNDP2 COG2 CPB2 CPS1 CPT1A CPT2 CREBBP CRH CSN1S1 CTGF CTSG CTSS CYP11A1 CYP27A1 CYP2C18 CYP2C19 CYP2C8 CYP2E1 CYP2J2 CYP3A43 CYP3A5 CYP3A7 CYP46A1 CYP4A11 CYP7A1 CYP7B1 CYSLTR1 CYSLTR2 DARS DDAH1 DDC DHCR24 DHCR7 DLG1 EDN1 EDN2 EDN3 EDNRA EEF1E1 ERAP1 ERN1 ERN2 F2 F2R F2RL1 F2RL2 F2RL3 FAAH FAAH2 FABP1 FABP2 FABP3 FABP4 FABP7 FADS1 FADS2 FDFT1 FDX1 FFAR1 FFAR2 FGF2 FGF21 FGFR4 FGR FHL2 FLT1 FPR2 G0S2 GAMT GATM GCG GCH1 GGCT GHSR GIP GLIPR1 GLTP GNA14 GNA15 GNAQ GNAT2 GNRH1 GNRH2 GNRHR GPR17 GPR65 GPRC6A GRK2 GRP GRPR GUCY1A3 GUCY1B3 HAL HARS2 HCRTR1 HCRTR2 HDAC3 HDC HDLBP HFE2 HMGA1 HMGCR HMGCS1 HMGCS2 HSD3B7 HSPG2 HTR2B IGF1 IL4I1 IL5 INSIG2 IRF6 IVL KARS KCNJ2 KCNJ4 KISS1 KISS1R KITLG KNG1 LARS LCMT2 LDLRAP1 LEP LIPA LIPE LMNA LNPEP LPAR2 LPAR3 LPAR4 LPAR6 LPL LRP8 LYZ MARS MC4R MCHR1 MCHR2 MDH2 ME1 MED1 MED10 MED11 MED13 MED13L MED14 MED15 MED16 MED17 MED18 MED19 MED20 MED21 MED23 MED25 MED26 MED27 MED29 MED30 MED4 MED6 MED7 MED8 METTL2B MLNR MMP14 MTTP MYH1 NAGS NCOR1 NFKB1 NFYA NFYB NFYC NMBR NMU NMUR1 NMUR2 NPAS2 NPC1 NPC1L1 NPFF NPFFR1 NPFFR2 NR1H2 NR3C1 NRIP1 NTS NTSR1 NTSR2 OAT OLR1 OPN4 OTC OXT OXTR P2RY1 P2RY10 P2RY2 P2RY6 PAPPA PCNA PCSK1 PCSK9 PDIA3 PEX11A PIK3CA PIK3CB PIK3R1 PIK3R2 PLA2G10 PLA2G12A PLA2G12B PLA2G1B PLA2G2D PLA2G2F PLA2G3 PLA2G4A PLA2G4B PLA2G5 PLB1 PLCB1 PLCB2 PLCB3 PLCB4 PLD1 PLD2 PLIN2 PLTP PMP2 PPARGC1A PRKCE PROK1 PROK2 PROKR1 PROKR2 PSMA2 PSMA3 PSMA4 PSMA5 PSMA6 PSMB1 PSMB2 PSMB4 PSMB5 PSMB6 PSMB7 PSMC1 PSMC2 PSMC3 PSMC4 PSMC5 PSMC6 PSMD10 PSMD11 PSMD14 PSMD4 PSMD5 PSMD7 PSME1 PSME2 PSME3 PSME4 PTAFR PTGDS PTGER1 PTGFR PTGIR PTGIS PTPN11 PTPN6 PYY QARS RAC1 RAC2 RARA RARS RARS2 RBP4 REN RETN RGL1 RGS19 RGS2 RHO RPN2 SC5D SCAP SCD SCD5 SCNN1G SDC1 SEC61A1 SEC61B SEC61G SELP SIN3A SIRT6 SLC15A3 SLC15A4 SLC16A10 SLC1A1 SLC27A1 SLC27A2 SLC27A4 SLC27A6 SLC38A4 SLC38A5 SLC3A1 SLC7A1 SLC7A3 SLC7A4 SLC7A5 SLC7A6 SLC7A7 SLC7A9 SMAD2 SMARCD3 SMO SREBF1 SST STARD5 STAT3 STS SULT2B1 TAC1 TAC3 TACR2 TACR3 TBL1X TBL1XR1 TEAD2 TEAD3 TGS1 TIAM2 TNFRSF21 TPP2 TRH TRHR TRIB3 TRPA1 TRPM3 TTR UCP1 UCP2 UCP3 USF1 UTS2R XCL1 XCL2 XCR1 YAP1 ZMYM6 ACOT1 ANXA6 ARHGEF25 ATP2A1 ATP5O AVPR1B CCK CEBPD DMD FFAR4 GAST GCGR GM2A GNG2 GNRHR2 GPR132 GRK5 HLA-C HMGB1 IARS INS INSIG1 KCNJ12 LCAT LCMT1 LPA LTB4R MME NCOA6 NMB NPS NPSR1 P4HB PIK3R3 PLA2G2A PLA2G6 PLIN5 PMCH PPIA PSMB10 PSMB11 PSMB3 PSMD12 PSMD13 QRFP SELL SLC22A4 SLC6A14 SRC SREBF2 TEAD1 TEAD4 TXNIP UGT1A9 ZDHHC17 ORC2 SLC25A2 RPS27A UBB UBC UBA52 HLA-A HLA-B ORC1 SLC25A15 GNA11 EPRS ART4 DOK1 GPR26 LPAR1 SPR TACR1 LAT2 SLC7A8 UCN2 UTS2 HCRT PPOX PSMD2 RPN1 GPR4 GPR19 RGS13 RGS18 C6 PSMA7 BAP1 MAGI1 TF H3F3A H3F3B RAC3 CKB CKM SLC22A5 HIST1H4H HIST1H4A HIST1H4B HIST1H4C HIST1H4D HIST1H4E HIST1H4F HIST1H4I HIST1H4J HIST1H4K HIST1H4L HIST2H4A HIST4H4 ACAT1 SOAT1 ANGPTL2 ANGPTL4 CEL FAP LIPC LIPH STAR SCARB1 ACAT2 SOAT2 ROR2 RORA ROR1 EP300 AHRR BPNT1 PIP SULT2A1 ST2 ACSBG1 HBG1 MED9 CLOCK SP1 MED22 MED24 TAZ WWTR1 NCOA3 TRAM1 MED12 OPA1 GRIP1 SCARB2 L1CAM PFN1 PRF1 CALM1 CALM2 EPO TIMP1 GDA PAEP CLGN HGF DAP IAPP TYR TYRL IGF2R CHAT CES2 CES1 HRAS KRAS |

**Supplementary table2 Arrhythmia-related genes and network**

| NCBI database | mapping String database | network in Cytoscape |
| --- | --- | --- |
| SCN5A | SCN5A | SCN5A |
| CDKN1A | CDKN1A | CDKN1A |
| IL6 | IL6 | IL6 |
| CRP | CRP | CRP |
| NOS3 | NOS3 | NOS3 |
| CFTR | CFTR | CFTR |
| SLC6A4 | SLC6A4 | SLC6A4 |
| KCNH2 | KCNH2 | KCNH2 |
| GNB5 | GNB5 | GNB5 |
| NPPB | NPPB | NPPB |
| CXCL12 | CXCL12 | CXCL12 |
| LMNA | LMNA | LMNA |
| EDN1 | EDN1 | EDN1 |
| KCNQ1 | KCNQ1 | KCNQ1 |
| LGALS3 | LGALS3 | LGALS3 |
| AGTR1 | AGTR1 | AGTR1 |
| ANK2 | ANK2 | ANK2 |
| DRD4 | DRD4 | DRD4 |
| GJA1 | GJA1 | GJA1 |
| COL1A1 | COL1A1 | COL1A1 |
| TTR | TTR | TTR |
| HRAS | HRAS | HRAS |
| SCN10A | SCN10A | SCN10A |
| MMP3 | MMP3 | MMP3 |
| FGF23 | FGF23 | FGF23 |
| CALM1 | CALM1 | CALM1 |
| RYR2 | RYR2 | RYR2 |
| ADM | ADM | ADM |
| CACNA1C | CACNA1C | CACNA1C |
| RYR1 | RYR1 | RYR1 |
| ADRB1 | ADRB1 | ADRB1 |
| CRYAB | CRYAB | CRYAB |
| NFIA | NFIA | NFIA |
| CHGA | CHGA | CHGA |
| MYH7 | MYH7 | MYH7 |
| GLA | GLA | GLA |
| KCNJ2 | KCNJ2 | KCNJ2 |
| APLN | APLN | APLN |
| NR3C2 | NR3C2 | NR3C2 |
| COL4A1 | COL4A1 | COL4A1 |
| DRD1 | DRD1 | DRD1 |
| MYBPC3 | MYBPC3 | MYBPC3 |
| PTK2B | PTK2B | PTK2B |
| SLC22A5 | SLC22A5 | - |
| KCNE1 | KCNE1 | KCNE1 |
| SCN4A | SCN4A | SCN4A |
| NKX2-5 | NKX2-5 | NKX2-5 |
| VCL | VCL | VCL |
| MAPK9 | MAPK9 | MAPK9 |
| KCNJ5 | KCNJ5 | KCNJ5 |
| BIN1 | BIN1 | BIN1 |
| DES | DES | DES |
| MIR1-1 | - | - |
| CAV3 | CAV3 | CAV3 |
| CXADR | CXADR | - |
| CAMK2G | CAMK2G | CAMK2G |
| KCNA5 | KCNA5 | KCNA5 |
| FLNC | FLNC | FLNC |
| CALM3 | CALM3 | CALM3 |
| PKP2 | PKP2 | PKP2 |
| SLC8A1 | SLC8A1 | SLC8A1 |
| HCN4 | HCN4 | HCN4 |
| FBLN1 | FBLN1 | FBLN1 |
| ATP2B4 | ATP2B4 | ATP2B4 |
| GJA5 | GJA5 | GJA5 |
| ST2 | SULT2A1 | SULT2A1 |
| CALM2 | CALM2 | CALM2 |
| KCNE2 | KCNE2 | KCNE2 |
| CACNA1S | CACNA1S | CACNA1S |
| MYL2 | MYL2 | MYL2 |
| SPTBN1 | SPTBN1 | SPTBN1 |
| ACTN2 | ACTN2 | ACTN2 |
| CEP85L | CEP85L | - |
| CASQ2 | CASQ2 | CASQ2 |
| PDE4A | PDE4A | PDE4A |
| SCN1B | SCN1B | SCN1B |
| AKAP9 | AKAP9 | AKAP9 |
| MIR19B1 | - | - |
| KCND3 | KCND3 | KCND3 |
| PDE3A | PDE3A | PDE3A |
| ECHS1 | ECHS1 | - |
| SNTA1 | SNTA1 | SNTA1 |
| MIR1-2 | - | - |
| KCNT1 | KCNT1 | KCNT1 |
| PEX7 | PEX7 | PEX7 |
| SLC5A7 | SLC5A7 | - |
| MYOT | MYOT | MYOT |
| FKBP1B | FKBP1B | FKBP1B |
| AKAP10 | AKAP10 | AKAP10 |
| PHYH | PHYH | PHYH |
| GABRA6 | GABRA6 | GABRA6 |
| LDB3 | LDB3 | LDB3 |
| MIR208A | - | - |
| GALR2 | GALR2 | GALR2 |
| TSPYL2 | TSPYL2 | - |
| CHST3 | CHST3 | - |
| MYL4 | MYL4 | MYL4 |
| SCN4B | SCN4B | SCN4B |
| KCNAB2 | KCNAB2 | KCNAB2 |
| TANGO2 | TANGO2 | - |
| LRRC10 | LRRC10 | LRRC10 |
| ALG10B | ALG10B | ALG10B |

**Supplementary table3 GO biological processes of ZT**

| Term | Count | Genes | Bonferroni |
| --- | --- | --- | --- |
| GO:0006936~muscle contraction | 7 | RYR1, GJA1, GALR2, CALM3, CACNA1S, CALM1, CALM2 | 7.454E-06 |
| GO:0001975~response to amphetamine | 5 | CALM3, DRD1, CALM1, CALM2, DRD4 | 8.533E-05 |
| GO:0007190~activation of adenylate cyclase activity | 5 | CALM3, ADRB1, DRD1, CALM1, CALM2 | 0.0002455 |
| GO:0019722~calcium-mediated signaling | 5 | EDN1, AGTR1, CALM3, CALM1, CALM2 | 0.0006634 |
| GO:0051412~response to corticosterone | 4 | CDKN1A, CALM3, CALM1, CALM2 | 0.001635 |
| GO:0030801~positive regulation of cyclic nucleotide metabolic process | 3 | CALM3, CALM1, CALM2 | 0.0042609 |
| GO:0050999~regulation of nitric-oxide synthase activity | 4 | NOS3, CALM3, CALM1, CALM2 | 0.0051579 |
| GO:0034220~ion transmembrane transport | 6 | RYR1, GJA1, GABRA6, CALM3, CALM1, CALM2 | 0.0095272 |
| GO:0002027~regulation of heart rate | 4 | CALM3, SCN5A, CALM1, CALM2 | 0.0107161 |
| GO:0051343~positive regulation of cyclic-nucleotide phosphodiesterase activity | 3 | CALM3, CALM1, CALM2 | 0.014106 |
| GO:1901841~regulation of high voltage-gated calcium channel activity | 3 | CALM3, CALM1, CALM2 | 0.0293381 |
| GO:0043647~inositol phosphate metabolic process | 4 | GALR2, CALM3, CALM1, CALM2 | 0.03107 |
| GO:1901844~regulation of cell communication by electrical coupling involved in cardiac conduction | 3 | CALM3, CALM1, CALM2 | 0.0388889 |
| GO:0060316~positive regulation of ryanodine-sensitive calcium-release channel activity | 3 | CALM3, CALM1, CALM2 | 0.049674 |
| GO:0042493~response to drug | 6 | COL1A1, IL6, CDKN1A, PDE3A, DRD1, SLC6A4 | 0.0544391 |
| GO:0051592~response to calcium ion | 4 | IL6, CALM3, CALM1, CALM2 | 0.0576595 |
| GO:0060315~negative regulation of ryanodine-sensitive calcium-release channel activity | 3 | CALM3, CALM1, CALM2 | 0.0889388 |
| GO:0032516~positive regulation of phosphoprotein phosphatase activity | 3 | CALM3, CALM1, CALM2 | 0.1041456 |
| GO:0010801~negative regulation of peptidyl-threonine phosphorylation | 3 | CALM3, CALM1, CALM2 | 0.1203108 |
| GO:0005513~detection of calcium ion | 3 | CALM3, CALM1, CALM2 | 0.1203108 |
| GO:0060307~regulation of ventricular cardiac muscle cell membrane repolarization | 3 | KCNH2, GJA1, SCN5A | 0.1738994 |
| GO:0010880~regulation of release of sequestered calcium ion into cytosol by sarcoplasmic reticulum | 3 | CALM3, CALM1, CALM2 | 0.1932311 |
| GO:0010881~regulation of cardiac muscle contraction by regulation of the release of sequestered calcium ion | 3 | CALM3, CALM1, CALM2 | 0.2131797 |
| GO:0060314~regulation of ryanodine-sensitive calcium-release channel activity | 3 | CALM3, CALM1, CALM2 | 0.2131797 |
| GO:0055117~regulation of cardiac muscle contraction | 3 | CALM3, CALM1, CALM2 | 0.2546409 |
| GO:0035307~positive regulation of protein dephosphorylation | 3 | CALM3, CALM1, CALM2 | 0.2546409 |
| GO:0031954~positive regulation of protein autophosphorylation | 3 | CALM3, CALM1, CALM2 | 0.2546409 |
| GO:0005980~glycogen catabolic process | 3 | CALM3, CALM1, CALM2 | 0.2546409 |
| GO:0032465~regulation of cytokinesis | 3 | CALM3, CALM1, CALM2 | 0.2760103 |
| GO:0051000~positive regulation of nitric-oxide synthase activity | 3 | CALM3, CALM1, CALM2 | 0.2760103 |
| GO:0010800~positive regulation of peptidyl-threonine phosphorylation | 3 | CALM3, CALM1, CALM2 | 0.3864505 |
| GO:0051482~positive regulation of cytosolic calcium ion concentration involved in phospholipase C-activating G-protein coupled signaling pathway | 3 | EDN1, AGTR1, DRD1 | 0.4087913 |
| GO:0086010~membrane depolarization during action potential | 3 | KCNH2, CACNA1S, SCN5A | 0.4087913 |
| GO:0043388~positive regulation of DNA binding | 3 | CALM3, CALM1, CALM2 | 0.4087913 |
| GO:0045909~positive regulation of vasodilation | 3 | GJA1, NOS3, APLN | 0.4310707 |
| GO:0022400~regulation of rhodopsin mediated signaling pathway | 3 | CALM3, CALM1, CALM2 | 0.4310707 |
| GO:0007204~positive regulation of cytosolic calcium ion concentration | 4 | EDN1, GJA1, GALR2, AGTR1 | 0.5026219 |
| GO:0000086~G2/M transition of mitotic cell cycle | 4 | CDKN1A, CALM3, CALM1, CALM2 | 0.5250171 |
| GO:0071902~positive regulation of protein serine/threonine kinase activity | 3 | CALM3, CALM1, CALM2 | 0.5604561 |
| GO:0007223~Wnt signaling pathway, calcium modulating pathway | 3 | CALM3, CALM1, CALM2 | 0.6394049 |
| GO:0061337~cardiac conduction | 3 | KCNH2, CACNA1S, SCN5A | 0.7421481 |
| GO:0071560~cellular response to transforming growth factor beta stimulus | 3 | COL1A1, EDN1, PDE3A | 0.7989558 |
| GO:0021762~substantia nigra development | 3 | CALM3, CALM1, CALM2 | 0.7989558 |
| GO:0042596~fear response | 2 | ADRB1, DRD4 | 0.9434737 |
| GO:0010628~positive regulation of gene expression | 4 | CRP, IL6, GJA1, SLC6A4 | 0.9903915 |
| GO:0003100~regulation of systemic arterial blood pressure by endothelin | 2 | EDN1, NOS3 | 0.9916767 |
| GO:0071321~cellular response to cGMP | 2 | PDE3A, SLC6A4 | 0.9968064 |
| GO:0060373~regulation of ventricular cardiac muscle cell membrane depolarization | 2 | GJA1, SCN5A | 0.9987747 |
| GO:0002576~platelet degranulation | 3 | CALM3, CALM1, CALM2 | 0.9989268 |
| GO:0010888~negative regulation of lipid storage | 2 | CRP, IL6 | 0.9995299 |
| GO:0060371~regulation of atrial cardiac muscle cell membrane depolarization | 2 | GJA1, SCN5A | 0.9995299 |
| GO:0071356~cellular response to tumor necrosis factor | 3 | COL1A1, IL6, EDN1 | 0.9995723 |
| GO:0086014~atrial cardiac muscle cell action potential | 2 | GJA1, SCN5A | 0.9998197 |
| GO:0034405~response to fluid shear stress | 2 | GJA1, NOS3 | 0.9998197 |
| GO:0042053~regulation of dopamine metabolic process | 2 | DRD1, DRD4 | 0.9999308 |
| GO:0046888~negative regulation of hormone secretion | 2 | IL6, EDN1 | 0.9999308 |
| GO:0042417~dopamine metabolic process | 2 | DRD1, DRD4 | 0.9999735 |
| GO:0031000~response to caffeine | 2 | RYR1, IL6 | 0.9999898 |
| GO:0030818~negative regulation of cAMP biosynthetic process | 2 | EDN1, DRD4 | 0.9999961 |
| GO:0001963~synaptic transmission, dopaminergic | 2 | DRD1, DRD4 | 0.9999961 |
| GO:0048148~behavioral response to cocaine | 2 | DRD1, DRD4 | 0.9999985 |
| GO:0086005~ventricular cardiac muscle cell action potential | 2 | KCNH2, SCN5A | 0.9999994 |
| GO:0055093~response to hyperoxia | 2 | COL1A1, CDKN1A | 0.9999999 |
| GO:0042310~vasoconstriction | 2 | EDN1, SLC6A4 | 0.9999999 |
| GO:0001659~temperature homeostasis | 2 | ADRB1, DRD1 | 1 |
| GO:0019229~regulation of vasoconstriction | 2 | EDN1, AGTR1 | 1 |
| GO:0038095~Fc-epsilon receptor signaling pathway | 3 | CALM3, CALM1, CALM2 | 1 |
| GO:0030335~positive regulation of cell migration | 3 | COL1A1, EDN1, DRD1 | 1 |
| GO:0001701~in utero embryonic development | 3 | EDN1, GJA1, NOS3 | 1 |
| GO:0007194~negative regulation of adenylate cyclase activity | 2 | GALR2, DRD4 | 1 |
| GO:0051899~membrane depolarization | 2 | EDN1, SCN5A | 1 |
| GO:0019228~neuronal action potential | 2 | DRD1, SCN5A | 1 |
| GO:0001662~behavioral fear response | 2 | DRD1, DRD4 | 1 |
| GO:2000379~positive regulation of reactive oxygen species metabolic process | 2 | CDKN1A, AGTR1 | 1 |
| GO:0045776~negative regulation of blood pressure | 2 | NOS3, APLN | 1 |
| GO:0043200~response to amino acid | 2 | IL6, EDN1 | 1 |
| GO:0007186~G-protein coupled receptor signaling pathway | 5 | EDN1, AGTR1, CALM3, CALM1, CALM2 | 1 |
| GO:0007585~respiratory gaseous exchange | 2 | EDN1, CFTR | 1 |
| GO:0086091~regulation of heart rate by cardiac conduction | 2 | KCNH2, SCN5A | 1 |
| GO:0009409~response to cold | 2 | IL6, ADRB1 | 1 |
| GO:0043547~positive regulation of GTPase activity | 4 | CALM3, ADRB1, CALM1, CALM2 | 1 |
| GO:0007267~cell-cell signaling | 3 | EDN1, GJA1, ADRB1 | 1 |
| GO:0006953~acute-phase response | 2 | CRP, IL6 | 1 |
| GO:0030819~positive regulation of cAMP biosynthetic process | 2 | ADRB1, DRD1 | 1 |
| GO:0000165~MAPK cascade | 3 | CALM3, CALM1, CALM2 | 1 |
| GO:0045429~positive regulation of nitric oxide biosynthetic process | 2 | IL6, EDN1 | 1 |
| GO:0043434~response to peptide hormone | 2 | COL1A1, GJA1 | 1 |
| GO:0060048~cardiac muscle contraction | 2 | KCNH2, SCN5A | 1 |
| GO:0051591~response to cAMP | 2 | COL1A1, PDE3A | 1 |
| GO:0035176~social behavior | 2 | DRD4, SLC6A4 | 1 |
| GO:0009408~response to heat | 2 | IL6, NOS3 | 1 |
| GO:0007189~adenylate cyclase-activating G-protein coupled receptor signaling pathway | 2 | GALR2, DRD1 | 1 |
| GO:0071277~cellular response to calcium ion | 2 | EDN1, SCN5A | 1 |
| GO:0016049~cell growth | 2 | IL6, EDN1 | 1 |
| GO:0051259~protein oligomerization | 2 | GJA1, SLC6A4 | 1 |
| GO:0048661~positive regulation of smooth muscle cell proliferation | 2 | IL6, EDN1 | 1 |
| GO:0050679~positive regulation of epithelial cell proliferation | 2 | IL6, SCN5A | 1 |
| GO:0045669~positive regulation of osteoblast differentiation | 2 | IL6, GJA1 | 1 |
| GO:0007613~memory | 2 | DRD1, SLC6A4 | 1 |
| GO:0030574~collagen catabolic process | 2 | COL1A1, MMP3 | 1 |
| GO:0008217~regulation of blood pressure | 2 | EDN1, NOS3 | 1 |
| GO:0007200~phospholipase C-activating G-protein coupled receptor signaling pathway | 2 | GALR2, AGTR1 | 1 |

**Supplementary table4 GO biological processes of GLT**

| Term | Count | Genes | Bonferroni |
| --- | --- | --- | --- |
| GO:0001975~response to amphetamine | 5 | CALM3, DRD1, CALM1, CALM2, DRD4 | 0.000202 |
| GO:0000165~MAPK cascade | 8 | ACTN2, AKAP9, PTK2B, CALM3, CALM1, HRAS, CALM2, SPTBN1 | 0.000278 |
| GO:0007190~activation of adenylate cyclase activity | 5 | CALM3, ADRB1, DRD1, CALM1, CALM2 | 0.00058 |
| GO:0006936~muscle contraction | 6 | GJA1, GALR2, CALM3, CACNA1S, CALM1, CALM2 | 0.000996 |
| GO:0006936~response to calcium ion | 5 | IL6, PTK2B, CALM3, CALM1, CALM2 | 0.002629 |
| GO:0060307~regulation of ventricular cardiac muscle cell membrane repolarization | 4 | KCNH2, GJA1, AKAP9, SCN5A | 0.002661 |
| GO:0051412~response to corticosterone | 4 | CDKN1A, CALM3, CALM1, CALM2 | 0.003189 |
| GO:0055117~regulation of cardiac muscle contraction | 4 | CALM3, CALM1, NKX2-5, CALM2 | 0.005172 |
| GO:0051000~positive regulation of nitric-oxide synthase activity | 4 | PTK2B, CALM3, CALM1, CALM2 | 0.005979 |
| GO:0030801~positive regulation of cyclic nucleotide metabolic process | 3 | CALM3, CALM1, CALM2 | 0.006895 |
| GO:0050999~regulation of nitric-oxide synthase activity | 4 | NOS3, CALM3, CALM1, CALM2 | 0.010024 |
| GO:0042493~response to drug | 7 | COL1A1, IL6, CDKN1A, PDE3A, PTK2B, DRD1, SLC6A4 | 0.011725 |
| GO:0045909~positive regulation of vasodilation | 4 | GJA1, NOS3, ADM, APLN | 0.014007 |
| GO:0002027~regulation of heart rate | 4 | CALM3, SCN5A, CALM1, CALM2 | 0.020739 |
| GO:0051343~positive regulation of cyclic-nucleotide phosphodiesterase activity | 3 | CALM3, CALM1, CALM2 | 0.022747 |
| GO:0043547~positive regulation of GTPase activity | 8 | ACTN2, AKAP9, CALM3, ADRB1, CALM1, HRAS, CALM2, SPTBN1 | 0.041936 |
| GO:1901841~regulation of high voltage-gated calcium channel activity | 3 | CALM3, CALM1, CALM2 | 0.047066 |
| GO:0061337~cardiac conduction | 4 | KCNH2, AKAP9, CACNA1S, SCN5A | 0.052279 |
| GO:0043647~inositol phosphate metabolic process | 4 | GALR2, CALM3, CALM1, CALM2 | 0.05937 |
| GO:1901844~regulation of cell communication by electrical coupling involved in cardiac conduction | 3 | CALM3, CALM1, CALM2 | 0.062189 |
| GO:0000086~G2/M transition of mitotic cell cycle | 5 | CDKN1A, AKAP9, CALM3, CALM1, CALM2 | 0.075696 |
| GO:0060316~positive regulation of ryanodine-sensitive calcium-release channel activity | 3 | CALM3, CALM1, CALM2 | 0.079149 |
| GO:0043267~negative regulation of potassium ion transport | 3 | ACTN2, NOS3, PTK2B | 0.097832 |
| GO:0060315~negative regulation of ryanodine-sensitive calcium-release channel activity | 3 | CALM3, CALM1, CALM2 | 0.139858 |
| GO:0032516~positive regulation of phosphoprotein phosphatase activity | 3 | CALM3, CALM1, CALM2 | 0.162931 |
| GO:0010801~negative regulation of peptidyl-threonine phosphorylation | 3 | CALM3, CALM1, CALM2 | 0.187188 |
| GO:0005513~detection of calcium ion | 3 | CALM3, CALM1, CALM2 | 0.187188 |
| GO:0010880~regulation of release of sequestered calcium ion into cytosol by sarcoplasmic reticulum | 3 | CALM3, CALM1, CALM2 | 0.293122 |
| GO:0060314~regulation of ryanodine-sensitive calcium-release channel activity | 3 | CALM3, CALM1, CALM2 | 0.321095 |
| GO:0010881~regulation of cardiac muscle contraction by regulation of the release of sequestered calcium ion | 3 | CALM3, CALM1, CALM2 | 0.321095 |
| GO:0031954~positive regulation of protein autophosphorylation | 3 | CALM3, CALM1, CALM2 | 0.377827 |
| GO:0035307~positive regulation of protein dephosphorylation | 3 | CALM3, CALM1, CALM2 | 0.377827 |
| GO:0005980~glycogen catabolic process | 3 | CALM3, CALM1, CALM2 | 0.377827 |
| GO:0008285~negative regulation of cell proliferation | 6 | IL6, CDKN1A, NOS3, PTK2B, ADM, HRAS | 0.399708 |
| GO:0032465~regulation of cytokinesis | 3 | CALM3, CALM1, CALM2 | 0.406316 |
| GO:0007194~negative regulation of adenylate cyclase activity | 3 | GALR2, AKAP9, DRD4 | 0.434718 |
| GO:0002576~platelet degranulation | 4 | ACTN2, CALM3, CALM1, CALM2 | 0.463517 |
| GO:0010800~positive regulation of peptidyl-threonine phosphorylation | 3 | CALM3, CALM1, CALM2 | 0.545191 |
| GO:0086010~membrane depolarization during action potential | 3 | KCNH2, CACNA1S, SCN5A | 0.571535 |
| GO:0043388~positive regulation of DNA binding | 3 | CALM3, CALM1, CALM2 | 0.571535 |
| GO:0022400~regulation of rhodopsin mediated signaling pathway | 3 | CALM3, CALM1, CALM2 | 0.597203 |
| GO:0010628~positive regulation of gene expression | 5 | CRP, IL6, GJA1, HRAS, SLC6A4 | 0.599994 |
| GO:0008284~positive regulation of cell proliferation | 6 | IL6, PTK2B, ADM, NKX2-5, HRAS, APLN | 0.652183 |
| GO:0086091~regulation of heart rate by cardiac conduction | 3 | KCNH2, AKAP9, SCN5A | 0.733895 |
| GO:0071902~positive regulation of protein serine/threonine kinase activity | 3 | CALM3, CALM1, CALM2 | 0.733895 |
| GO:0007204~positive regulation of cytosolic calcium ion concentration | 4 | GJA1, GALR2, PTK2B, ADM | 0.735244 |
| GO:0009409~response to cold | 3 | IL6, ADM, ADRB1 | 0.753454 |
| GO:0007223~Wnt signaling pathway, calcium modulating pathway | 3 | CALM3, CALM1, CALM2 | 0.806317 |
| GO:0030819~positive regulation of cAMP biosynthetic process | 3 | ADM, ADRB1, DRD1 | 0.822014 |
| GO:0060048~cardiac muscle contraction | 3 | KCNH2, SCN5A, NKX2-5 | 0.886837 |
| GO:0051591~response to cAMP | 3 | COL1A1, PDE3A, PTK2B | 0.897256 |
| GO:0035176~social behavior | 3 | HRAS, DRD4, SLC6A4 | 0.915807 |
| GO:0021762~substantia nigra development | 3 | CALM3, CALM1, CALM2 | 0.924012 |
| GO:0019722~calcium-mediated signaling | 3 | CALM3, CALM1, CALM2 | 0.938466 |
| GO:0038095~Fc-epsilon receptor signaling pathway | 4 | CALM3, CALM1, HRAS, CALM2 | 0.948648 |
| GO:0007165~signal transduction | 8 | GJA1, AKAP9, PDE4A, PTK2B, ADM, HRAS, NR3C2, APLN | 0.95709 |
| GO:0030335~positive regulation of cell migration | 4 | COL1A1, PTK2B, DRD1, HRAS | 0.961521 |
| GO:0050679~positive regulation of epithelial cell proliferation | 3 | IL6, SCN5A, HRAS | 0.978372 |
| GO:0042596~fear response | 2 | ADRB1, DRD4 | 0.979363 |
| GO:0034220~ion transmembrane transport | 4 | GJA1, CALM3, CALM1, CALM2 | 0.990936 |
| GO:0045906~negative regulation of vasoconstriction | 2 | ADM, APLN | 0.99434 |
| GO:0071300~cellular response to retinoic acid | 3 | COL1A1, PTK2B, SLC6A4 | 0.994351 |
| GO:0043066~negative regulation of apoptotic process | 5 | IL6, CDKN1A, PDE3A, PTK2B, NKX2-5 | 0.998634 |
| GO:0050830~defense response to Gram-positive bacterium | 3 | CRP, IL6, ADM | 0.999466 |
| GO:1901018~positive regulation of potassium ion transmembrane transporter activity | 2 | ACTN2, AKAP9 | 0.999574 |
| GO:0071321~cellular response to cGMP | 2 | PDE3A, SLC6A4 | 0.999574 |
| GO:0060373~regulation of ventricular cardiac muscle cell membrane depolarization | 2 | GJA1, SCN5A | 0.999883 |
| GO:0031175~neuron projection development | 3 | IL6, GALR2, PTK2B | 0.999966 |
| GO:0060371~regulation of atrial cardiac muscle cell membrane depolarization | 2 | GJA1, SCN5A | 0.999968 |
| GO:0010888~negative regulation of lipid storage | 2 | CRP, IL6 | 0.999968 |
| GO:0086014~atrial cardiac muscle cell action potential | 2 | GJA1, SCN5A | 0.999991 |
| GO:0034405~response to fluid shear stress | 2 | GJA1, NOS3 | 0.999991 |
| GO:0043268~positive regulation of potassium ion transport | 2 | ACTN2, DRD1 | 0.999998 |
| GO:0042053~regulation of dopamine metabolic process | 2 | DRD1, DRD4 | 0.999998 |
| GO:0045766~positive regulation of angiogenesis | 3 | NOS3, PTK2B, ADM | 0.999999 |
| GO:0042417~dopamine metabolic process | 2 | DRD1, DRD4 | 0.999999 |
| GO:0043116~negative regulation of vascular permeability | 2 | PDE3A, ADM | 1 |
| GO:0060306~regulation of membrane repolarization | 2 | KCNH2, AKAP9 | 1 |
| GO:0007512~adult heart development | 2 | GJA1, NKX2-5 | 1 |
| GO:0001878~response to yeast | 2 | IL6, ADM | 1 |
| GO:0001963~synaptic transmission, dopaminergic | 2 | DRD1, DRD4 | 1 |
| GO:0048148~behavioral response to cocaine | 2 | DRD1, DRD4 | 1 |
| GO:0010629~negative regulation of gene expression | 3 | CDKN1A, GJA1, HRAS | 1 |
| GO:0006198~cAMP catabolic process | 2 | PDE3A, PDE4A | 1 |
| GO:0086005~ventricular cardiac muscle cell action potential | 2 | KCNH2, SCN5A | 1 |
| GO:0060292~long term synaptic depression | 2 | PTK2B, DRD1 | 1 |
| GO:0055093~response to hyperoxia | 2 | COL1A1, CDKN1A | 1 |
| GO:0001556~oocyte maturation | 2 | PDE3A, PTK2B | 1 |
| GO:0030500~regulation of bone mineralization | 2 | GJA1, PTK2B | 1 |
| GO:0051968~positive regulation of synaptic transmission, glutamatergic | 2 | PTK2B, DRD1 | 1 |
| GO:0010765~positive regulation of sodium ion transport | 2 | SCN5A, NKX2-5 | 1 |
| GO:0042832~defense response to protozoan | 2 | IL6, HRAS | 1 |
| GO:0001659~temperature homeostasis | 2 | ADRB1, DRD1 | 1 |
| GO:0002026~regulation of the force of heart contraction | 2 | ADM, APLN | 1 |
| GO:0001666~response to hypoxia | 3 | PTK2B, ADM, SLC6A4 | 1 |
| GO:0070374~positive regulation of ERK1 and ERK2 cascade | 3 | IL6, PTK2B, HRAS | 1 |
| GO:0090398~cellular senescence | 2 | CDKN1A, HRAS | 1 |
| GO:0051602~response to electrical stimulus | 2 | IL6, AKAP9 | 1 |
| GO:0048041~focal adhesion assembly | 2 | ACTN2, PTK2B | 1 |
| GO:0007507~heart development | 3 | GJA1, ADM, NKX2-5 | 1 |
| GO:0019228~neuronal action potential | 2 | DRD1, SCN5A | 1 |
| GO:0001662~behavioral fear response | 2 | DRD1, DRD4 | 1 |
| GO:0045214~sarcomere organization | 2 | ACTN2, NKX2-5 | 1 |
| GO:0097193~intrinsic apoptotic signaling pathway | 2 | CDKN1A, HRAS | 1 |
| GO:0045776~negative regulation of blood pressure | 2 | NOS3, APLN | 1 |
| GO:0045765~regulation of angiogenesis | 2 | IL6, PTK2B | 1 |
| GO:0019933~cAMP-mediated signaling | 2 | PDE3A, ADM | 1 |
| GO:0060291~long-term synaptic potentiation | 2 | PTK2B, DRD1 | 1 |
| GO:0006953~acute-phase response | 2 | CRP, IL6 | 1 |
| GO:0007268~chemical synaptic transmission | 3 | GALR2, AKAP9, SLC6A4 | 1 |
| GO:0045740~positive regulation of DNA replication | 2 | IL6, HRAS | 1 |
| GO:0045429~positive regulation of nitric oxide biosynthetic process | 2 | IL6, PTK2B | 1 |
| GO:0043434~response to peptide hormone | 2 | COL1A1, GJA1 | 1 |
| GO:0007267~cell-cell signaling | 3 | GJA1, ADM, ADRB1 | 1 |
| GO:0045860~positive regulation of protein kinase activity | 2 | CDKN1A, PTK2B | 1 |
| GO:0031100~organ regeneration | 2 | CDKN1A, ADM | 1 |
| GO:0009408~response to heat | 2 | IL6, NOS3 | 1 |
| GO:0071560~cellular response to transforming growth factor beta stimulus | 2 | COL1A1, PDE3A | 1 |
| GO:0007189~adenylate cyclase-activating G-protein coupled receptor signaling pathway | 2 | GALR2, DRD1 | 1 |
| GO:0042542~response to hydrogen peroxide | 2 | COL1A1, PTK2B | 1 |
| GO:0007166~cell surface receptor signaling pathway | 3 | GALR2, PTK2B, HRAS | 1 |
| GO:0071320~cellular response to cAMP | 2 | AKAP9, CFTR | 1 |
| GO:0045727~positive regulation of translation | 2 | IL6, PTK2B | 1 |
| GO:0050829~defense response to Gram-negative bacterium | 2 | IL6, ADM | 1 |
| GO:0042475~odontogenesis of dentin-containing tooth | 2 | ADM, SCN5A | 1 |
| GO:0007173~epidermal growth factor receptor signaling pathway | 2 | PTK2B, HRAS | 1 |
| GO:0001570~vasculogenesis | 2 | ADM, NKX2-5 | 1 |

**Supplementary table5 GO biological processes of HET**

| Term | Count | Genes | Bonferroni |
| --- | --- | --- | --- |
| GO:0002027~regulation of heart rate | 5 | CAV3, CALM3, SCN5A, CALM1, CALM2 | 8.35E-05 |
| GO:0007190~activation of adenylate cyclase activity | 5 | CALM3, ADRB1, DRD1, CALM1, CALM2 | 0.000185 |
| GO:0019722~calcium-mediated signaling | 5 | EDN1, AGTR1, CALM3, CALM1, CALM2 | 0.000501 |
| GO:0060307~regulation of ventricular cardiac muscle cell membrane repolarization | 4 | KCNH2, GJA1, CAV3, SCN5A | 0.001125 |
| GO:0051412~response to corticosterone | 4 | CDKN1A, CALM3, CALM1, CALM2 | 0.001349 |
| GO:0055117~regulation of cardiac muscle contraction | 4 | CAV3, CALM3, CALM1, CALM2 | 0.002192 |
| GO:0030801~positive regulation of cyclic nucleotide metabolic process | 3 | CALM3, CALM1, CALM2 | 0.00383 |
| GO:0050999~regulation of nitric-oxide synthase activity | 4 | NOS3, CALM3, CALM1, CALM2 | 0.00426 |
| GO:0001975~response to amphetamine | 4 | CALM3, DRD1, CALM1, CALM2 | 0.007319 |
| GO:0006936~muscle contraction | 5 | GJA1, CAV3, CALM3, CALM1, CALM2 | 0.009768 |
| GO:0051343~positive regulation of cyclic-nucleotide phosphodiesterase activity | 3 | CALM3, CALM1, CALM2 | 0.012689 |
| GO:1901841~regulation of high voltage-gated calcium channel activity | 3 | CALM3, CALM1, CALM2 | 0.026416 |
| GO:0060373~regulation of ventricular cardiac muscle cell membrane depolarization | 3 | GJA1, CAV3, SCN5A | 0.026416 |
| GO:1901844~regulation of cell communication by electrical coupling involved in cardiac conduction | 3 | CALM3, CALM1, CALM2 | 0.035036 |
| GO:0042493~response to drug | 6 | COL1A1, IL6, CDKN1A, PDE3A, DRD1, SLC6A4 | 0.038662 |
| GO:0060316~positive regulation of ryanodine-sensitive calcium-release channel activity | 3 | CALM3, CALM1, CALM2 | 0.04478 |
| GO:0051592~response to calcium ion | 4 | IL6, CALM3, CALM1, CALM2 | 0.047976 |
| GO:0060315~negative regulation of ryanodine-sensitive calcium-release channel activity | 3 | CALM3, CALM1, CALM2 | 0.080362 |
| GO:0032516~positive regulation of phosphoprotein phosphatase activity | 3 | CALM3, CALM1, CALM2 | 0.094186 |
| GO:0010801~negative regulation of peptidyl-threonine phosphorylation | 3 | CALM3, CALM1, CALM2 | 0.10891 |
| GO:0005513~detection of calcium ion | 3 | CALM3, CALM1, CALM2 | 0.10891 |
| GO:0086005~ventricular cardiac muscle cell action potential | 3 | KCNH2, CAV3, SCN5A | 0.124478 |
| GO:0010880~regulation of release of sequestered calcium ion into cytosol by sarcoplasmic reticulum | 3 | CALM3, CALM1, CALM2 | 0.175689 |
| GO:0060314~regulation of ryanodine-sensitive calcium-release channel activity | 3 | CALM3, CALM1, CALM2 | 0.194066 |
| GO:0010881~regulation of cardiac muscle contraction by regulation of the release of sequestered calcium ion | 3 | CALM3, CALM1, CALM2 | 0.194066 |
| GO:0031954~positive regulation of protein autophosphorylation | 3 | CALM3, CALM1, CALM2 | 0.232416 |
| GO:0035307~positive regulation of protein dephosphorylation | 3 | CALM3, CALM1, CALM2 | 0.232416 |
| GO:0005980~glycogen catabolic process | 3 | CALM3, CALM1, CALM2 | 0.232416 |
| GO:0032465~regulation of cytokinesis | 3 | CALM3, CALM1, CALM2 | 0.252268 |
| GO:0051000~positive regulation of nitric-oxide synthase activity | 3 | CALM3, CALM1, CALM2 | 0.252268 |
| GO:0010628~positive regulation of gene expression | 5 | CRP, IL6, GJA1, HRAS, SLC6A4 | 0.270075 |
| GO:0010800~positive regulation of peptidyl-threonine phosphorylation | 3 | CALM3, CALM1, CALM2 | 0.35587 |
| GO:0051482~positive regulation of cytosolic calcium ion concentration involved in phospholipase C-activating G-protein coupled signaling pathway | 3 | EDN1, AGTR1, DRD1 | 0.37705 |
| GO:0043388~positive regulation of DNA binding | 3 | CALM3, CALM1, CALM2 | 0.37705 |
| GO:0022400~regulation of rhodopsin mediated signaling pathway | 3 | CALM3, CALM1, CALM2 | 0.398253 |
| GO:0007204~positive regulation of cytosolic calcium ion concentration | 4 | EDN1, GJA1, CAV3, AGTR1 | 0.441234 |
| GO:0000086~G2/M transition of mitotic cell cycle | 4 | CDKN1A, CALM3, CALM1, CALM2 | 0.462366 |
| GO:0086091~regulation of heart rate by cardiac conduction | 3 | KCNH2, SCN5A, KCNJ2 | 0.523177 |
| GO:0071902~positive regulation of protein serine/threonine kinase activity | 3 | CALM3, CALM1, CALM2 | 0.523177 |
| GO:0007223~Wnt signaling pathway, calcium modulating pathway | 3 | CALM3, CALM1, CALM2 | 0.601183 |
| GO:0061337~cardiac conduction | 3 | KCNH2, SCN5A, KCNJ2 | 0.705368 |
| GO:0038095~Fc-epsilon receptor signaling pathway | 4 | CALM3, CALM1, HRAS, CALM2 | 0.732132 |
| GO:0043647~inositol phosphate metabolic process | 3 | CALM3, CALM1, CALM2 | 0.736089 |
| GO:0071560~cellular response to transforming growth factor beta stimulus | 3 | COL1A1, EDN1, PDE3A | 0.764682 |
| GO:0021762~substantia nigra development | 3 | CALM3, CALM1, CALM2 | 0.764682 |
| GO:0030335~positive regulation of cell migration | 4 | COL1A1, EDN1, DRD1, HRAS | 0.764915 |
| GO:0016049~cell growth | 3 | IL6, EDN1, CAV3 | 0.847982 |
| GO:0034220~ion transmembrane transport | 4 | GJA1, CALM3, CALM1, CALM2 | 0.878274 |
| GO:0050679~positive regulation of epithelial cell proliferation | 3 | IL6, SCN5A, HRAS | 0.884446 |
| GO:0071260~cellular response to mechanical stimulus | 3 | COL1A1, GJA1, KCNJ2 | 0.950354 |
| GO:0000165~MAPK cascade | 4 | CALM3, CALM1, HRAS, CALM2 | 0.980006 |
| GO:0003100~regulation of systemic arterial blood pressure by endothelin | 2 | EDN1, NOS3 | 0.990698 |
| GO:0086011~membrane repolarization during action potential | 2 | KCNH2, KCNJ2 | 0.990698 |
| GO:0043547~positive regulation of GTPase activity | 5 | CALM3, ADRB1, CALM1, HRAS, CALM2 | 0.994363 |
| GO:0071321~cellular response to cGMP | 2 | PDE3A, SLC6A4 | 0.996351 |
| GO:0002576~platelet degranulation | 3 | CALM3, CALM1, CALM2 | 0.997947 |
| GO:0071356~cellular response to tumor necrosis factor | 3 | COL1A1, IL6, EDN1 | 0.99911 |
| GO:1901380~negative regulation of potassium ion transmembrane transport | 2 | KCNH2, CAV3 | 0.999438 |
| GO:0060371~regulation of atrial cardiac muscle cell membrane depolarization | 2 | GJA1, SCN5A | 0.999438 |
| GO:0010888~negative regulation of lipid storage | 2 | CRP, IL6 | 0.999438 |
| GO:0034405~response to fluid shear stress | 2 | GJA1, NOS3 | 0.99978 |
| GO:0086014~atrial cardiac muscle cell action potential | 2 | GJA1, SCN5A | 0.99978 |
| GO:0046888~negative regulation of hormone secretion | 2 | IL6, EDN1 | 0.999914 |
| GO:0086013~membrane repolarization during cardiac muscle cell action potential | 2 | KCNH2, KCNJ2 | 0.999914 |
| GO:0010629~negative regulation of gene expression | 3 | CDKN1A, GJA1, HRAS | 0.999977 |
| GO:0060306~regulation of membrane repolarization | 2 | KCNH2, KCNJ2 | 0.999987 |
| GO:0086004~regulation of cardiac muscle cell contraction | 2 | SCN5A, KCNJ2 | 0.999987 |
| GO:0008285~negative regulation of cell proliferation | 4 | IL6, CDKN1A, NOS3, HRAS | 0.999994 |
| GO:0086012~membrane depolarization during cardiac muscle cell action potential | 2 | SCN5A, KCNJ2 | 0.999995 |
| GO:0051926~negative regulation of calcium ion transport | 2 | CAV3, NOS3 | 0.999995 |
| GO:1901381~positive regulation of potassium ion transmembrane transport | 2 | KCNH2, KCNJ2 | 0.999999 |
| GO:0086002~cardiac muscle cell action potential involved in contraction | 2 | SCN5A, KCNJ2 | 1 |
| GO:0055093~response to hyperoxia | 2 | COL1A1, CDKN1A | 1 |
| GO:0042310~vasoconstriction | 2 | EDN1, SLC6A4 | 1 |
| GO:0042832~defense response to protozoan | 2 | IL6, HRAS | 1 |
| GO:0046716~muscle cell cellular homeostasis | 2 | IL6, CAV3 | 1 |
| GO:0001659~temperature homeostasis | 2 | ADRB1, DRD1 | 1 |
| GO:0008284~positive regulation of cell proliferation | 4 | IL6, EDN1, CAV3, HRAS | 1 |
| GO:0019229~regulation of vasoconstriction | 2 | EDN1, AGTR1 | 1 |
| GO:0001701~in utero embryonic development | 3 | EDN1, GJA1, NOS3 | 1 |
| GO:0090398~cellular senescence | 2 | CDKN1A, HRAS | 1 |
| GO:0051899~membrane depolarization | 2 | EDN1, SCN5A | 1 |
| GO:0007186~G-protein coupled receptor signaling pathway | 5 | EDN1, AGTR1, CALM3, CALM1, CALM2 | 1 |
| GO:0019228~neuronal action potential | 2 | DRD1, SCN5A | 1 |
| GO:0086010~membrane depolarization during action potential | 2 | KCNH2, SCN5A | 1 |
| GO:0045909~positive regulation of vasodilation | 2 | GJA1, NOS3 | 1 |
| GO:0097193~intrinsic apoptotic signaling pathway | 2 | CDKN1A, HRAS | 1 |
| GO:2000379~positive regulation of reactive oxygen species metabolic process | 2 | CDKN1A, AGTR1 | 1 |
| GO:0034504~protein localization to nucleus | 2 | COL1A1, LMNA | 1 |
| GO:0043200~response to amino acid | 2 | IL6, EDN1 | 1 |
| GO:0009409~response to cold | 2 | IL6, ADRB1 | 1 |
| GO:0007267~cell-cell signaling | 3 | EDN1, GJA1, ADRB1 | 1 |
| GO:0006953~acute-phase response | 2 | CRP, IL6 | 1 |
| GO:0030819~positive regulation of cAMP biosynthetic process | 2 | ADRB1, DRD1 | 1 |
| GO:0045740~positive regulation of DNA replication | 2 | IL6, HRAS | 1 |
| GO:0045429~positive regulation of nitric oxide biosynthetic process | 2 | IL6, EDN1 | 1 |
| GO:0043434~response to peptide hormone | 2 | COL1A1, GJA1 | 1 |
| GO:0060048~cardiac muscle contraction | 2 | KCNH2, SCN5A | 1 |
| GO:0051591~response to cAMP | 2 | COL1A1, PDE3A | 1 |
| GO:0009408~response to heat | 2 | IL6, NOS3 | 1 |
| GO:0035176~social behavior | 2 | HRAS, SLC6A4 | 1 |
| GO:0071277~cellular response to calcium ion | 2 | EDN1, SCN5A | 1 |
| GO:0051259~protein oligomerization | 2 | GJA1, SLC6A4 | 1 |
| GO:0043406~positive regulation of MAP kinase activity | 2 | EDN1, HRAS | 1 |
| GO:0048661~positive regulation of smooth muscle cell proliferation | 2 | IL6, EDN1 | 1 |
| GO:0045669~positive regulation of osteoblast differentiation | 2 | IL6, GJA1 | 1 |
| GO:0007613~memory | 2 | DRD1, SLC6A4 | 1 |
| GO:0030574~collagen catabolic process | 2 | COL1A1, MMP3 | 1 |
| GO:0008217~regulation of blood pressure | 2 | EDN1, NOS3 | 1 |
| GO:0035690~cellular response to drug | 2 | KCNH2, EDN1 | 1 |
| GO:0007265~Ras protein signal transduction | 2 | CDKN1A, HRAS | 1 |
| GO:0071300~cellular response to retinoic acid | 2 | COL1A1, SLC6A4 | 1 |
| GO:0071347~cellular response to interleukin-1 | 2 | IL6, EDN1 | 1 |

**Supplementary table6 KEGG pathways of ZT**

| Term | Count | Genes | Bonferroni |
| --- | --- | --- | --- |
| hsa04924:Renin secretion | 7 | PDE3A, AGTR1, CALM3, ADRB1, CACNA1S, CALM1, CALM2 | 5.01117E-06 |
| hsa04020:Calcium signaling pathway | 9 | RYR1, NOS3, AGTR1, CALM3, ADRB1, DRD1, CACNA1S, CALM1, CALM2 | 6.9468E-06 |
| hsa04022:cGMP-PKG signaling pathway | 8 | NOS3, PDE3A, AGTR1, CALM3, ADRB1, CACNA1S, CALM1, CALM2 | 5.84566E-05 |
| hsa04024:cAMP signaling pathway | 8 | PDE3A, CALM3, ADRB1, DRD1, CACNA1S, CALM1, CALM2, CFTR | 0.000268404 |
| hsa04261:Adrenergic signaling in cardiomyocytes | 7 | AGTR1, CALM3, ADRB1, CACNA1S, SCN5A, CALM1, CALM2 | 0.000490095 |
| hsa04921:Oxytocin signaling pathway | 7 | RYR1, CDKN1A, NOS3, CALM3, CACNA1S, CALM1, CALM2 | 0.000794654 |
| hsa04925:Aldosterone synthesis and secretion | 5 | AGTR1, CALM3, CACNA1S, CALM1, CALM2 | 0.014551679 |
| hsa04270:Vascular smooth muscle contraction | 5 | AGTR1, CALM3, CACNA1S, CALM1, CALM2 | 0.058534564 |
| hsa04728:Dopaminergic synapse | 5 | CALM3, DRD1, CALM1, CALM2, DRD4 | 0.08122153 |
| hsa05214:Glioma | 4 | CDKN1A, CALM3, CALM1, CALM2 | 0.128157049 |
| hsa05031:Amphetamine addiction | 4 | CALM3, DRD1, CALM1, CALM2 | 0.133564844 |
| hsa04971:Gastric acid secretion | 4 | CALM3, CALM1, CALM2, CFTR | 0.174656279 |
| hsa05133:Pertussis | 4 | IL6, CALM3, CALM1, CALM2 | 0.187395534 |
| hsa04080:Neuroactive ligand-receptor interaction | 6 | GABRA6, GALR2, AGTR1, ADRB1, DRD1, DRD4 | 0.187890285 |
| hsa04970:Salivary secretion | 4 | CALM3, ADRB1, CALM1, CALM2 | 0.264492233 |
| hsa04912:GnRH signaling pathway | 4 | CALM3, CACNA1S, CALM1, CALM2 | 0.302882362 |
| hsa04744:Phototransduction | 3 | CALM3, CALM1, CALM2 | 0.32506885 |
| hsa04713:Circadian entrainment | 4 | RYR1, CALM3, CALM1, CALM2 | 0.334758017 |
| hsa04066:HIF-1 signaling pathway | 4 | IL6, CDKN1A, EDN1, NOS3 | 0.342862137 |
| hsa04915:Estrogen signaling pathway | 4 | NOS3, CALM3, CALM1, CALM2 | 0.367446001 |
| hsa04916:Melanogenesis | 4 | EDN1, CALM3, CALM1, CALM2 | 0.375719829 |
| hsa05010:Alzheimer's disease | 4 | CALM3, CACNA1S, CALM1, CALM2 | 0.859745506 |
| hsa04720:Long-term potentiation | 3 | CALM3, CALM1, CALM2 | 0.893417441 |
| hsa05034:Alcoholism | 4 | CALM3, DRD1, CALM1, CALM2 | 0.895426296 |
| hsa05152:Tuberculosis | 4 | IL6, CALM3, CALM1, CALM2 | 0.895426296 |
| hsa04540:Gap junction | 3 | GJA1, ADRB1, DRD1 | 0.978725039 |
| hsa05032:Morphine addiction | 3 | GABRA6, PDE3A, DRD1 | 0.983404211 |
| hsa04070:Phosphatidylinositol signaling system | 3 | CALM3, CALM1, CALM2 | 0.990937381 |
| hsa04750:Inflammatory mediator regulation of TRP channels | 3 | CALM3, CALM1, CALM2 | 0.990937381 |
| hsa04922:Glucagon signaling pathway | 3 | CALM3, CALM1, CALM2 | 0.991711472 |
| hsa04668:TNF signaling pathway | 3 | IL6, EDN1, MMP3 | 0.996043075 |
| hsa04114:Oocyte meiosis | 3 | CALM3, CALM1, CALM2 | 0.997310551 |
| hsa04722:Neurotrophin signaling pathway | 3 | CALM3, CALM1, CALM2 | 0.99891487 |
| hsa04910:Insulin signaling pathway | 3 | CALM3, CALM1, CALM2 | 0.999848631 |

**Supplementary table7 KEGG pathways of GLT**

| Term | Count | Genes | Bonferroni |
| --- | --- | --- | --- |
| hsa04024:cAMP signaling pathway | 9 | PDE3A, PDE4A, CALM3, ADRB1, DRD1, CACNA1S, CALM1, CALM2, CFTR | 2.86E-05 |
| hsa04020:Calcium signaling pathway | 8 | NOS3, PTK2B, CALM3, ADRB1, DRD1, CACNA1S, CALM1, CALM2 | 0.000241 |
| hsa04924:Renin secretion | 6 | PDE3A, CALM3, ADRB1, CACNA1S, CALM1, CALM2 | 0.000311 |
| hsa04921:Oxytocin signaling pathway | 7 | CDKN1A, NOS3, CALM3, CACNA1S, CALM1, HRAS, CALM2 | 0.001333 |
| hsa04912:GnRH signaling pathway | 6 | PTK2B, CALM3, CACNA1S, CALM1, HRAS, CALM2 | 0.001781 |
| hsa04022:cGMP-PKG signaling pathway | 7 | NOS3, PDE3A, CALM3, ADRB1, CACNA1S, CALM1, CALM2 | 0.001797 |
| hsa05214:Glioma | 5 | CDKN1A, CALM3, CALM1, HRAS, CALM2 | 0.009395 |
| hsa04261:Adrenergic signaling in cardiomyocytes | 6 | CALM3, ADRB1, CACNA1S, SCN5A, CALM1, CALM2 | 0.013233 |
| hsa04915:Estrogen signaling pathway | 5 | NOS3, CALM3, CALM1, HRAS, CALM2 | 0.047329 |
| hsa04728:Dopaminergic synapse | 5 | CALM3, DRD1, CALM1, CALM2, DRD4 | 0.12054 |
| hsa05031:Amphetamine addiction | 4 | CALM3, DRD1, CALM1, CALM2 | 0.188288 |
| hsa04720:Long-term potentiation | 4 | CALM3, CALM1, HRAS, CALM2 | 0.188288 |
| hsa04971:Gastric acid secretion | 4 | CALM3, CALM1, CALM2, CFTR | 0.24354 |
| hsa05133:Pertussis | 4 | IL6, CALM3, CALM1, CALM2 | 0.260411 |
| hsa04925:Aldosterone synthesis and secretion | 4 | CALM3, CACNA1S, CALM1, CALM2 | 0.313442 |
| hsa05034:Alcoholism | 5 | CALM3, DRD1, CALM1, HRAS, CALM2 | 0.346138 |
| hsa04970:Salivary secretion | 4 | CALM3, ADRB1, CALM1, CALM2 | 0.359861 |
| hsa04540:Gap junction | 4 | GJA1, ADRB1, DRD1, HRAS | 0.378846 |
| hsa04744:Phototransduction | 3 | CALM3, CALM1, CALM2 | 0.422191 |
| hsa04916:Melanogenesis | 4 | CALM3, CALM1, HRAS, CALM2 | 0.494998 |
| hsa04270:Vascular smooth muscle contraction | 4 | CALM3, CACNA1S, CALM1, CALM2 | 0.653073 |
| hsa04722:Neurotrophin signaling pathway | 4 | CALM3, CALM1, HRAS, CALM2 | 0.678719 |
| hsa04910:Insulin signaling pathway | 4 | CALM3, CALM1, HRAS, CALM2 | 0.811005 |
| hsa05161:Hepatitis B | 4 | IL6, CDKN1A, PTK2B, HRAS | 0.851281 |
| hsa05010:Alzheimer's disease | 4 | CALM3, CACNA1S, CALM1, CALM2 | 0.940993 |
| hsa05152:Tuberculosis | 4 | IL6, CALM3, CALM1, CALM2 | 0.961245 |
| hsa04151:PI3K-Akt signaling pathway | 5 | COL1A1, IL6, CDKN1A, NOS3, HRAS | 0.985992 |
| hsa04015:Rap1 signaling pathway | 4 | CALM3, CALM1, HRAS, CALM2 | 0.993798 |
| hsa05032:Morphine addiction | 3 | PDE3A, PDE4A, DRD1 | 0.996626 |
| hsa04014:Ras signaling pathway | 4 | CALM3, CALM1, HRAS, CALM2 | 0.997842 |
| hsa04713:Circadian entrainment | 3 | CALM3, CALM1, CALM2 | 0.997897 |
| hsa04066:HIF-1 signaling pathway | 3 | IL6, CDKN1A, NOS3 | 0.998136 |
| hsa04750:Inflammatory mediator regulation of TRP channels | 3 | CALM3, CALM1, CALM2 | 0.998539 |
| hsa04070:Phosphatidylinositol signaling system | 3 | CALM3, CALM1, CALM2 | 0.998539 |
| hsa04922:Glucagon signaling pathway | 3 | CALM3, CALM1, CALM2 | 0.998709 |
| hsa05146:Amoebiasis | 3 | COL1A1, IL6, ACTN2 | 0.999471 |
| hsa04726:Serotonergic synapse | 3 | CACNA1S, HRAS, SLC6A4 | 0.999728 |
| hsa04114:Oocyte meiosis | 3 | CALM3, CALM1, CALM2 | 0.999728 |
| hsa04080:Neuroactive ligand-receptor interaction | 4 | GALR2, ADRB1, DRD1, DRD4 | 0.999964 |
| hsa04068:FoxO signaling pathway | 3 | IL6, CDKN1A, HRAS | 0.99999 |

**Supplementary table8 KEGG pathways of HET**

| Term | Count | Genes | Bonferroni |
| --- | --- | --- | --- |
| hsa04924:Renin secretion | 7 | PDE3A, AGTR1, CALM3, ADRB1, CALM1, CALM2, KCNJ2 | 2.93E-06 |
| hsa04921:Oxytocin signaling pathway | 7 | CDKN1A, NOS3, CALM3, CALM1, HRAS, CALM2, KCNJ2 | 0.000475 |
| hsa04022:cGMP-PKG signaling pathway | 7 | NOS3, PDE3A, AGTR1, CALM3, ADRB1, CALM1, CALM2 | 0.000642 |
| hsa04020:Calcium signaling pathway | 7 | NOS3, AGTR1, CALM3, ADRB1, DRD1, CALM1, CALM2 | 0.00132 |
| hsa05214:Glioma | 5 | CDKN1A, CALM3, CALM1, HRAS, CALM2 | 0.004535 |
| hsa04261:Adrenergic signaling in cardiomyocytes | 6 | AGTR1, CALM3, ADRB1, SCN5A, CALM1, CALM2 | 0.005587 |
| hsa04915:Estrogen signaling pathway | 5 | NOS3, CALM3, CALM1, HRAS, CALM2 | 0.023346 |
| hsa04916:Melanogenesis | 5 | EDN1, CALM3, CALM1, HRAS, CALM2 | 0.024259 |
| hsa04024:cAMP signaling pathway | 6 | PDE3A, CALM3, ADRB1, DRD1, CALM1, CALM2 | 0.030494 |
| hsa05031:Amphetamine addiction | 4 | CALM3, DRD1, CALM1, CALM2 | 0.11048 |
| hsa04720:Long-term potentiation | 4 | CALM3, CALM1, HRAS, CALM2 | 0.11048 |
| hsa04971:Gastric acid secretion | 4 | CALM3, CALM1, CALM2, KCNJ2 | 0.145277 |
| hsa05133:Pertussis | 4 | IL6, CALM3, CALM1, CALM2 | 0.156141 |
| hsa04925:Aldosterone synthesis and secretion | 4 | AGTR1, CALM3, CALM1, CALM2 | 0.191065 |
| hsa05034:Alcoholism | 5 | CALM3, DRD1, CALM1, HRAS, CALM2 | 0.191481 |
| hsa04970:Salivary secretion | 4 | CALM3, ADRB1, CALM1, CALM2 | 0.222673 |
| hsa04540:Gap junction | 4 | GJA1, ADRB1, DRD1, HRAS | 0.235902 |
| hsa04912:GnRH signaling pathway | 4 | CALM3, CALM1, HRAS, CALM2 | 0.256326 |
| hsa04066:HIF-1 signaling pathway | 4 | IL6, CDKN1A, EDN1, NOS3 | 0.291766 |
| hsa04744:Phototransduction | 3 | CALM3, CALM1, CALM2 | 0.296474 |
| hsa04270:Vascular smooth muscle contraction | 4 | AGTR1, CALM3, CALM1, CALM2 | 0.453049 |
| hsa04722:Neurotrophin signaling pathway | 4 | CALM3, CALM1, HRAS, CALM2 | 0.476771 |
| hsa04728:Dopaminergic synapse | 4 | CALM3, DRD1, CALM1, CALM2 | 0.539539 |
| hsa04910:Insulin signaling pathway | 4 | CALM3, CALM1, HRAS, CALM2 | 0.615329 |
| hsa05152:Tuberculosis | 4 | IL6, CALM3, CALM1, CALM2 | 0.848028 |
| hsa04151:PI3K-Akt signaling pathway | 5 | COL1A1, IL6, CDKN1A, NOS3, HRAS | 0.894629 |
| hsa04015:Rap1 signaling pathway | 4 | CALM3, CALM1, HRAS, CALM2 | 0.948769 |
| hsa04014:Ras signaling pathway | 4 | CALM3, CALM1, HRAS, CALM2 | 0.972756 |
| hsa04713:Circadian entrainment | 3 | CALM3, CALM1, CALM2 | 0.981893 |
| hsa04070:Phosphatidylinositol signaling system | 3 | CALM3, CALM1, CALM2 | 0.985753 |
| hsa04750:Inflammatory mediator regulation of TRP channels | 3 | CALM3, CALM1, CALM2 | 0.985753 |
| hsa04922:Glucagon signaling pathway | 3 | CALM3, CALM1, CALM2 | 0.986865 |
| hsa04668:TNF signaling pathway | 3 | IL6, EDN1, MMP3 | 0.993301 |
| hsa04114:Oocyte meiosis | 3 | CALM3, CALM1, CALM2 | 0.995289 |
| hsa04068:FoxO signaling pathway | 3 | IL6, CDKN1A, HRAS | 0.999485 |
| hsa05161:Hepatitis B | 3 | IL6, CDKN1A, HRAS | 0.99984 |
| hsa05202:Transcriptional misregulation in cancer | 3 | IL6, CDKN1A, MMP3 | 0.999987 |
| hsa05010:Alzheimer's disease | 3 | CALM3, CALM1, CALM2 | 0.999989 |

**Supplementary tables of ANOVA results**

| Occurance of VA |  |  | Sum of Squares | df | Mean Square | *F* | *P* |
| --- | --- | --- | --- | --- | --- | --- | --- |
| Between Groups | (Combined) |  | 1.540 | 3 | 0.513 | 1.310 | 0.306 |
|  | Linear Term | Contrast | 0.010 | 1 | 0.010 | 0.027 | 0.873 |
|  |  | Deviation | 1.529 | 2 | 0.765 | 1.951 | 0.174 |
| Within Groups |  |  | 6.270 | 16 | 0.392 |  |  |
| Total |  |  | 7.810 | 19 |  |  |  |

| Occurance of VA | (I) | (J) | Mean Difference (I-J) | Std. Error | *P* | 95% Confidence Interval | |
| --- | --- | --- | --- | --- | --- | --- | --- |
|  |  |  |  |  |  | Lower Bound | Upper Bound |
| LSD | Vehicle | ZT | -0.762 | 0.396 | 0.072 | -1.601 | 0.077 |
|  |  | GLT | -0.306 | 0.396 | 0.451 | -1.145 | 0.533 |
|  |  | HET | -0.220 | 0.396 | 0.586 | -1.059 | 0.619 |
|  | ZT | Vehicle | 0.762 | 0.396 | 0.072 | -0.077 | 1.601 |
|  |  | GLT | 0.456 | 0.396 | 0.266 | -0.383 | 1.295 |
|  |  | HET | 0.542 | 0.396 | 0.190 | -0.297 | 1.381 |
|  | GLT | Vehicle | 0.306 | 0.396 | 0.451 | -0.533 | 1.145 |
|  |  | ZT | -0.456 | 0.396 | 0.266 | -1.295 | 0.383 |
|  |  | HET | 0.086 | 0.396 | 0.831 | -0.753 | 0.925 |
|  | HET | Vehicle | 0.220 | 0.396 | 0.586 | -0.619 | 1.059 |
|  |  | ZT | -0.542 | 0.396 | 0.190 | -1.381 | 0.297 |
|  |  | GLT | -0.086 | 0.396 | 0.831 | -0.925 | 0.753 |

| Duration of VA |  |  | Sum of Squares | df | Mean Square | *F* | *P* |
| --- | --- | --- | --- | --- | --- | --- | --- |
| Between Groups | (Combined) |  | 343.284 | 3 | 114.428 | 84.087 | 0.000 |
|  | Linear Term | Contrast | 230.675 | 1 | 230.675 | 169.511 | 0.000 |
|  |  | Deviation | 112.609 | 2 | 56.304 | 41.375 | 0.000 |
| Within Groups |  |  | 21.773 | 16 | 1.361 |  |  |
| Total |  |  | 365.057 | 19 |  |  |  |

| Duration of VA | (I) | (J) | Mean Difference (I-J) | Std. Error | *P* | 95% Confidence Interval | |
| --- | --- | --- | --- | --- | --- | --- | --- |
|  |  |  |  |  |  | Lower Bound | Upper Bound |
| LSD | Vehicle | ZT | 9.39800* | 0.738 | 0.000 | 7.834 | 10.962 |
|  |  | GLT | 8.41200* | 0.738 | 0.000 | 6.848 | 9.976 |
|  |  | HET | 10.45400* | 0.738 | 0.000 | 8.890 | 12.018 |
|  | ZT | Vehicle | -9.39800* | 0.738 | 0.000 | -10.962 | -7.834 |
|  |  | GLT | -0.986 | 0.738 | 0.200 | -2.550 | 0.578 |
|  |  | HET | 1.056 | 0.738 | 0.172 | -0.508 | 2.620 |
|  | GLT | Vehicle | -8.41200* | 0.738 | 0.000 | -9.976 | -6.848 |
|  |  | ZT | 0.986 | 0.738 | 0.200 | -0.578 | 2.550 |
|  |  | HET | 2.04200* | 0.738 | 0.014 | 0.478 | 3.606 |
|  | HET | Vehicle | -10.45400* | 0.738 | 0.000 | -12.018 | -8.890 |
|  |  | ZT | -1.056 | 0.738 | 0.172 | -2.620 | 0.508 |
|  |  | GLT | -2.04200* | 0.738 | 0.014 | -3.606 | -0.478 |

| CALM1 |  |  | Sum of Squares | df | Mean Square | *F* | *P* |
| --- | --- | --- | --- | --- | --- | --- | --- |
| Between Groups | (Combined) |  | 17.299 | 4 | 4.325 | 15.935 | 0.000 |
|  | Linear Term | Contrast | 0.359 | 1 | 0.359 | 1.323 | 0.264 |
|  |  | Deviation | 16.940 | 3 | 5.647 | 20.805 | 0.000 |
| Within Groups |  |  | 5.428 | 20 | 0.271 |  |  |
| Total |  |  | 22.727 | 24 |  |  |  |

| CALM1 | (I) | (J) | Mean Difference (I-J) | Std. Error | *P* | 95% Confidence Interval | |
| --- | --- | --- | --- | --- | --- | --- | --- |
|  |  |  |  |  |  | Lower Bound | Upper Bound |
| LSD | Control | Vehicle | -2.44170* | 0.329 | 0.000 | -3.129 | -1.754 |
|  |  | ZT | -1.91573* | 0.329 | 0.000 | -2.603 | -1.228 |
|  |  | GLT | -1.29441* | 0.329 | 0.001 | -1.982 | -0.607 |
|  |  | HET | -.99735* | 0.329 | 0.007 | -1.685 | -0.310 |
|  | Vehicle | Control | 2.44170* | 0.329 | 0.000 | 1.754 | 3.129 |
|  |  | ZT | 0.526 | 0.329 | 0.126 | -0.161 | 1.213 |
|  |  | GLT | 1.14729* | 0.329 | 0.002 | 0.460 | 1.835 |
|  |  | HET | 1.44435* | 0.329 | 0.000 | 0.757 | 2.132 |
|  | ZT | Vehicle | 1.91573* | 0.329 | 0.000 | 1.228 | 2.603 |
|  |  | Control | -0.526 | 0.329 | 0.126 | -1.213 | 0.161 |
|  |  | GLT | 0.621 | 0.329 | 0.074 | -0.066 | 1.309 |
|  |  | HET | .91838* | 0.329 | 0.011 | 0.231 | 1.606 |
|  | GLT | Vehicle | 1.29441* | 0.329 | 0.001 | 0.607 | 1.982 |
|  |  | Control | -1.14729* | 0.329 | 0.002 | -1.835 | -0.460 |
|  |  | ZT | -0.621 | 0.329 | 0.074 | -1.309 | 0.066 |
|  |  | HET | 0.297 | 0.329 | 0.378 | -0.390 | 0.984 |
|  | HET | Vehicle | .99735* | 0.329 | 0.007 | 0.310 | 1.685 |
|  |  | Control | -1.44435* | 0.329 | 0.000 | -2.132 | -0.757 |
|  |  | ZT | -.91838* | 0.329 | 0.011 | -1.606 | -0.231 |
|  |  | GLT | -0.297 | 0.329 | 0.378 | -0.984 | 0.390 |

| CALM2 |  |  | Sum of Squares | df | Mean Square | *F* | *P* |
| --- | --- | --- | --- | --- | --- | --- | --- |
| Between Groups | (Combined) |  | 12.809 | 4 | 3.202 | 11.460 | 0.000 |
|  | Linear Term | Contrast | 0.825 | 1 | 0.825 | 2.954 | 0.101 |
|  |  | Deviation | 11.984 | 3 | 3.995 | 14.295 | 0.000 |
| Within Groups |  |  | 5.589 | 20 | 0.279 |  |  |
| Total |  |  | 18.398 | 24 |  |  |  |

| CALM2 | (I) | (J) | Mean Difference (I-J) | Std. Error | *P* | 95% Confidence Interval | |
| --- | --- | --- | --- | --- | --- | --- | --- |
|  |  |  |  |  |  | Lower Bound | Upper Bound |
| LSD | Control | Vehicle | -1.98411* | 0.334 | 0.000 | -2.682 | -1.287 |
|  |  | ZT | -0.68148 | 0.334 | 0.055 | -1.379 | 0.016 |
|  |  | GLT | -0.59254 | 0.334 | 0.092 | -1.290 | 0.105 |
|  |  | HET | -0.05333 | 0.334 | 0.875 | -0.751 | 0.644 |
|  | Vehicle | Control | 1.98411* | 0.334 | 0.000 | 1.287 | 2.682 |
|  |  | ZT | 1.30263* | 0.334 | 0.001 | 0.605 | 2.000 |
|  |  | GLT | 1.39157* | 0.334 | 0.000 | 0.694 | 2.089 |
|  |  | HET | 1.93078* | 0.334 | 0.000 | 1.233 | 2.628 |
|  | ZT | Vehicle | 0.68148 | 0.334 | 0.055 | -0.016 | 1.379 |
|  |  | Control | -1.30263* | 0.334 | 0.001 | -2.000 | -0.605 |
|  |  | GLT | 0.08894 | 0.334 | 0.793 | -0.609 | 0.786 |
|  |  | HET | 0.62815 | 0.334 | 0.075 | -0.069 | 1.326 |
|  | GLT | Vehicle | 0.59254 | 0.334 | 0.092 | -0.105 | 1.290 |
|  |  | Control | -1.39157* | 0.334 | 0.000 | -2.089 | -0.694 |
|  |  | ZT | -0.08894 | 0.334 | 0.793 | -0.786 | 0.609 |
|  |  | HET | 0.53921 | 0.334 | 0.122 | -0.158 | 1.237 |
|  | HET | Vehicle | 0.05333 | 0.334 | 0.875 | -0.644 | 0.751 |
|  |  | Control | -1.93078* | 0.334 | 0.000 | -2.628 | -1.233 |
|  |  | ZT | -0.62815 | 0.334 | 0.075 | -1.326 | 0.069 |
|  |  | GLT | -0.53921 | 0.334 | 0.122 | -1.237 | 0.158 |

| CALM3 |  |  | Sum of Squares | df | Mean Square | *F* | *P* |
| --- | --- | --- | --- | --- | --- | --- | --- |
| Between Groups | (Combined) |  | 6.818 | 4 | 1.704 | 6.100 | 0.002 |
|  | Linear Term | Contrast | 1.103 | 1 | 1.103 | 3.947 | 0.061 |
|  |  | Deviation | 5.715 | 3 | 1.905 | 6.818 | 0.002 |
| Within Groups |  |  | 5.588 | 20 | 0.279 |  |  |
| Total |  |  | 12.406 | 24 |  |  |  |

| CALM3 | (I) | (J) | Mean Difference (I-J) | Std. Error | *P* | 95% Confidence Interval | |
| --- | --- | --- | --- | --- | --- | --- | --- |
|  |  |  |  |  |  | Lower Bound | Upper Bound |
| LSD | Control | Vehicle | -1.50311* | 0.334 | 0.000 | -2.201 | -0.806 |
|  |  | ZT | -1.29375* | 0.334 | 0.001 | -1.991 | -0.596 |
|  |  | GLT | -1.14672* | 0.334 | 0.003 | -1.844 | -0.449 |
|  |  | HET | -.92080* | 0.334 | 0.012 | -1.618 | -0.223 |
|  | Vehicle | Control | 1.50311* | 0.334 | 0.000 | 0.806 | 2.201 |
|  |  | ZT | 0.209 | 0.334 | 0.538 | -0.488 | 0.907 |
|  |  | GLT | 0.356 | 0.334 | 0.299 | -0.341 | 1.054 |
|  |  | HET | 0.582 | 0.334 | 0.097 | -0.115 | 1.280 |
|  | ZT | Vehicle | 1.29375* | 0.334 | 0.001 | 0.596 | 1.991 |
|  |  | Control | -0.209 | 0.334 | 0.538 | -0.907 | 0.488 |
|  |  | GLT | 0.147 | 0.334 | 0.665 | -0.550 | 0.844 |
|  |  | HET | 0.373 | 0.334 | 0.278 | -0.324 | 1.070 |
|  | GLT | Vehicle | 1.14672* | 0.334 | 0.003 | 0.449 | 1.844 |
|  |  | Control | -0.356 | 0.334 | 0.299 | -1.054 | 0.341 |
|  |  | ZT | -0.147 | 0.334 | 0.665 | -0.844 | 0.550 |
|  |  | HET | 0.226 | 0.334 | 0.507 | -0.471 | 0.923 |
|  | HET | Vehicle | .92080* | 0.334 | 0.012 | 0.223 | 1.618 |
|  |  | Control | -0.582 | 0.334 | 0.097 | -1.280 | 0.115 |
|  |  | ZT | -0.373 | 0.334 | 0.278 | -1.070 | 0.324 |
|  |  | GLT | -0.226 | 0.334 | 0.507 | -0.923 | 0.471 |

| CRP |  |  | Sum of Squares | df | Mean Square | *F* | *P* |
| --- | --- | --- | --- | --- | --- | --- | --- |
| Between Groups | (Combined) |  | 9.735 | 4 | 2.434 | 5.144 | 0.005 |
|  | Linear Term | Contrast | 0.000 | 1 | 0.000 | 0.000 | 0.996 |
|  |  | Deviation | 9.735 | 3 | 3.245 | 6.858 | 0.002 |
| Within Groups |  |  | 9.463 | 20 | 0.473 |  |  |
| Total |  |  | 19.198 | 24 |  |  |  |

| CRP | (I) | (J) | Mean Difference (I-J) | Std. Error | *P* | 95% Confidence Interval | |
| --- | --- | --- | --- | --- | --- | --- | --- |
|  |  |  |  |  |  | Lower Bound | Upper Bound |
| LSD | Control | Vehicle | -1.69569* | 0.43504 | 0.001 | -2.6032 | -0.7882 |
|  |  | ZT | -1.37121* | 0.43504 | 0.005 | -2.2787 | -0.4637 |
|  |  | GLT | -.95172* | 0.43504 | 0.041 | -1.8592 | -0.0442 |
|  |  | HET | -0.36929 | 0.43504 | 0.406 | -1.2768 | 0.5382 |
|  | Vehicle | Control | 1.69569* | 0.43504 | 0.001 | 0.7882 | 2.6032 |
|  |  | ZT | 0.32448 | 0.43504 | 0.464 | -0.583 | 1.232 |
|  |  | GLT | 0.74397 | 0.43504 | 0.103 | -0.1635 | 1.6514 |
|  |  | HET | 1.32640* | 0.43504 | 0.006 | 0.4189 | 2.2339 |
|  | ZT | Vehicle | 1.37121* | 0.43504 | 0.005 | 0.4637 | 2.2787 |
|  |  | Control | -0.32448 | 0.43504 | 0.464 | -1.232 | 0.583 |
|  |  | GLT | 0.41949 | 0.43504 | 0.346 | -0.488 | 1.327 |
|  |  | HET | 1.00192* | 0.43504 | 0.032 | 0.0944 | 1.9094 |
|  | GLT | Vehicle | .95172* | 0.43504 | 0.041 | 0.0442 | 1.8592 |
|  |  | Control | -0.74397 | 0.43504 | 0.103 | -1.6514 | 0.1635 |
|  |  | ZT | -0.41949 | 0.43504 | 0.346 | -1.327 | 0.488 |
|  |  | HET | 0.58243 | 0.43504 | 0.196 | -0.3251 | 1.4899 |
|  | HET | Vehicle | 0.36929 | 0.43504 | 0.406 | -0.5382 | 1.2768 |
|  |  | Control | -1.32640* | 0.43504 | 0.006 | -2.2339 | -0.4189 |
|  |  | ZT | -1.00192* | 0.43504 | 0.032 | -1.9094 | -0.0944 |
|  |  | GLT | -0.58243 | 0.43504 | 0.196 | -1.4899 | 0.3251 |

| IL-6 |  |  | Sum of Squares | df | Mean Square | *F* | *P* |
| --- | --- | --- | --- | --- | --- | --- | --- |
| Between Groups | (Combined) |  | 9.646 | 4 | 2.412 | 14.101 | 0.000 |
|  | Linear Term | Contrast | 0.014 | 1 | 0.014 | 0.080 | 0.781 |
|  |  | Deviation | 9.633 | 3 | 3.211 | 18.775 | 0.000 |
| Within Groups |  |  | 3.420 | 20 | 0.171 |  |  |
| Total |  |  | 13.067 | 24 |  |  |  |

| IL-6 | (I) | (J) | Mean Difference (I-J) | Std. Error | *P* | 95% Confidence Interval | |
| --- | --- | --- | --- | --- | --- | --- | --- |
|  |  |  |  |  |  | Lower Bound | Upper Bound |
| LSD | Control | Vehicle | -1.86156* | 0.262 | 0.000 | -2.407 | -1.316 |
|  |  | ZT | -1.05523* | 0.262 | 0.001 | -1.601 | -0.510 |
|  |  | GLT | -.67759* | 0.262 | 0.017 | -1.223 | -0.132 |
|  |  | HET | -0.509 | 0.262 | 0.066 | -1.055 | 0.036 |
|  | Vehicle | Control | 1.86156* | 0.262 | 0.000 | 1.316 | 2.407 |
|  |  | ZT | .80633* | 0.262 | 0.006 | 0.261 | 1.352 |
|  |  | GLT | 1.18397* | 0.262 | 0.000 | 0.638 | 1.730 |
|  |  | HET | 1.35207* | 0.262 | 0.000 | 0.807 | 1.898 |
|  | ZT | Vehicle | 1.05523* | 0.262 | 0.001 | 0.510 | 1.601 |
|  |  | Control | -.80633* | 0.262 | 0.006 | -1.352 | -0.261 |
|  |  | GLT | 0.378 | 0.262 | 0.164 | -0.168 | 0.923 |
|  |  | HET | .54574* | 0.262 | 0.050 | 0.000 | 1.091 |
|  | GLT | Vehicle | .67759* | 0.262 | 0.017 | 0.132 | 1.223 |
|  |  | Control | -1.18397* | 0.262 | 0.000 | -1.730 | -0.638 |
|  |  | ZT | -0.378 | 0.262 | 0.164 | -0.923 | 0.168 |
|  |  | HET | 0.168 | 0.262 | 0.528 | -0.378 | 0.714 |
|  | HET | Vehicle | 0.509 | 0.262 | 0.066 | -0.036 | 1.055 |
|  |  | Control | -1.35207* | 0.262 | 0.000 | -1.898 | -0.807 |
|  |  | ZT | -.54574* | 0.262 | 0.050 | -1.091 | 0.000 |
|  |  | GLT | -0.168 | 0.262 | 0.528 | -0.714 | 0.378 |

| NOS3 |  |  | Sum of Squares | df | Mean Square | *F* | *P* |
| --- | --- | --- | --- | --- | --- | --- | --- |
| Between Groups | (Combined) |  | 5.701 | 4 | 1.425 | 5.531 | 0.004 |
|  | Linear Term | Contrast | 0.031 | 1 | 0.031 | 0.121 | 0.731 |
|  |  | Deviation | 5.670 | 3 | 1.890 | 7.334 | 0.002 |
| Within Groups |  |  | 5.154 | 20 | 0.258 |  |  |
| Total |  |  | 10.854 | 24 |  |  |  |

| NOS3 | (I) | (J) | Mean Difference (I-J) | Std. Error | *P* | 95% Confidence Interval | |
| --- | --- | --- | --- | --- | --- | --- | --- |
|  |  |  |  |  |  | Lower Bound | Upper Bound |
| LSD | Control | Vehicle | -1.36684* | 0.32105 | 0 | -2.0365 | -0.6971 |
|  |  | ZT | -.86248* | 0.32105 | 0.014 | -1.5322 | -0.1928 |
|  |  | GLT | -0.62319 | 0.32105 | 0.066 | -1.2929 | 0.0465 |
|  |  | HET | -0.24692 | 0.32105 | 0.451 | -0.9166 | 0.4228 |
|  | Vehicle | Control | 1.36684* | 0.32105 | 0 | 0.6971 | 2.0365 |
|  |  | ZT | 0.50436 | 0.32105 | 0.132 | -0.1653 | 1.1741 |
|  |  | GLT | .74365* | 0.32105 | 0.031 | 0.074 | 1.4133 |
|  |  | HET | 1.11992* | 0.32105 | 0.002 | 0.4502 | 1.7896 |
|  | ZT | Vehicle | .86248* | 0.32105 | 0.014 | 0.1928 | 1.5322 |
|  |  | Control | -0.50436 | 0.32105 | 0.132 | -1.1741 | 0.1653 |
|  |  | GLT | 0.23928 | 0.32105 | 0.465 | -0.4304 | 0.909 |
|  |  | HET | 0.61556 | 0.32105 | 0.07 | -0.0541 | 1.2853 |
|  | GLT | Vehicle | 0.62319 | 0.32105 | 0.066 | -0.0465 | 1.2929 |
|  |  | Control | -.74365* | 0.32105 | 0.031 | -1.4133 | -0.074 |
|  |  | ZT | -0.23928 | 0.32105 | 0.465 | -0.909 | 0.4304 |
|  |  | HET | 0.37628 | 0.32105 | 0.255 | -0.2934 | 1.046 |
|  | HET | Vehicle | 0.24692 | 0.32105 | 0.451 | -0.4228 | 0.9166 |
|  |  | Control | -1.11992* | 0.32105 | 0.002 | -1.7896 | -0.4502 |
|  |  | ZT | -0.61556 | 0.32105 | 0.07 | -1.2853 | 0.0541 |
|  |  | GLT | -0.37628 | 0.32105 | 0.255 | -1.046 | 0.2934 |
